# Screening of metabolic markers present in *Oxytropis* by UHPLC-Q-TOF/MS and preliminary pharmacophylogenetic investigation

**DOI:** 10.3389/fpls.2022.958460

**Published:** 2022-10-20

**Authors:** Xin Jia, Yang Liu, Suwei Wang, Jiannan Ma, Juan Yu, Xin Yue, Ying Zhang, Xiaoqin Wang

**Affiliations:** College of Pharmacy, Inner Mongolia Medical University, Hohhot, China

**Keywords:** chemotaxonomy, ethnopharmacology, metabolomics, pharmacophylogenetics, *Oxytropis*

## Abstract

Plants belonging to the *Oxytropis* genus, family Leguminosae, are found throughout the world, with about 80 species mainly distributed in northwest and northeast China. The plants have medicinal properties and many plants have been used as folk medicine for the treatment of colds, inflammation of carbuncle swelling, pain, and different types of bleeding. In recent years, due to the reduced availability of wild resources and increased clinical demand, additional *Oxytropis* species have been used in Mongolian medicine. This study explored the medicinal potential of four *Oxytropis* species, investigating their phylogeny, chemical components, and pharmacological activities. *Oxytropis myriophylla* (Pall) DC., *Oxytropis hirta* Bunge, and *Oxytropis bicolor* Bge. were found to be closely related at the taxonomic level. While previous investigations on the bioactive constituents of *Oxytropis* have been limited and have concentrated largely on flavonoids and saponins, the present study established a novel UHPLC-Q-TOF/MS based on metabolite profiling to comprehensively analyze the chemical composition of the four *Oxytropis* species and to identify marker compounds. A total of 75 compounds were identified from the four species, with 23 identified as characteristic marker components. Twenty-six marker compounds were identified in *O. myriophylla* from different geographical regions. Analysis of pharmacological activity showed that extracts of *O. myriophylla* and *O. hirta* had stronger anti-inflammatory activity than the extracts from the other species. The relationships between the chemical components, traditional curative uses, and pharmacological activities were analyzed to provide a preliminary documentation of the pharmacophylogenetic characteristics of the *Oxytropis* family as a whole. Several marker compounds, including licoricesaponin G2, licoricesaponin J2, and glycyrrhizic acid found in *O. hirta* were found to have effective anti-inflammatory activity, consistent with the traditional application of reducing swelling and healing wounds. This preliminary investigation into the pharmacophylogeny of the genus *Oxytropis* will contribute to the conservation and exploitation of the medicinal resources of this genus.

## Introduction

There are ~350 *Oxytropis* species (family Leguminosae) found throughout the world, of which about 80 species are mainly distributed in northwest and northeast China (Elisens and Denford, 1982; Sun and Xu, 1992). Many of these plants have been used as folk medicine for the treatment of colds, inflammation of carbuncle swelling, pain, and different types of bleeding (Batsuren et al., 1992). In recent years, due to the dwindling availability of wild resources and increasing clinical demand, additional *Oxitropis* species are being used in Mongolian medicine. Two species, in particular, *Oxytropis bicolor* Bunge and *Oxytropis racemosa* Turcz., are abundant and are often used as substitutes in clinical applications. All four *Oxytropis* species are widely distributed in Inner Mongolia, especially *O. myriophylla*, which is the most commonly used in medicinal applications and is prevalent in the eastern, central, and western regions of Inner Mongolia. Table 1 shows details of the ethnopharmacology of the four *Oxytropis* species, with information on geographical distribution, synonyms (common names), medicinal parts, traditional uses, and pharmacological activities, drawn from both herbal books and published literature.

**Table 1:** The distribution, traditional curative effect, and pharmacological activities of four *Oxytropis*.

**Plant**	**Synonyms/Common names**	**Distribution**	**Medicinal part**	**Traditional curative effect**	**Pharmacological activities**	**Ref**.
*O. bicolor*	“Rentoucao”, “Diding”, “Maozhuahua”	Inner Mongolia, Hebei, Shanxi, Shaanxi, Ningxia, Gansu, Qinghai, Henan	Seed	Detoxification and analgesia	Antibacterial	(Chen et al., 2005)
*O. hirta*	“Oxytropishirta Bunge”	Heilongjiang, Jilin, Liaoning, Inner Mongolia, Hebei, Shanxi, Shandong, Shaanxi, Gansu, Henan, Hubei	Whole grass	Kill “sticky”, clear away heat, dry “xieriwusu”, callus, regenerate muscle, lock pulse, stop bleeding, reduce swelling and defecate	Antibacterial	(Ye et al., 2022)
*O. myriophylla*	“*Oxytropis myriophylloides* Hurus.”	Inner Mongolia, Heilongjiang, Jilin, Liaoning, Hebei, Shaanxi, Shanxi, Ningxia	Whole grass	Kill “sticky”, clear away heat, dry “xieriwusu”, heal wounds, regenerate muscles, stop bleeding and reduce swelling	Anti inflammatory and analgesic, antibacterial, antioxidant	(She et al., 2010; Saiyin, 2014; Meng et al., 2016)
*O. racemosa*	“Paopaocao”, “Maozhuazhua” “Yazuidou”, “OxytropismandshuricaBge.”	Hebei, Shanxi, Inner Mongolia, Shaanxi, Gansu	Whole grass	Promoting digestion	Improve digestive function	(Zhang et al., 2019)

Despite their extensive usage, there has been extremely limited research on these *Oxytropis* species, especially in terms of their chemical composition. To date, the major bioactive constituents described in *Oxytropis* have been limited to flavonoids and saponins, with minimal investigation into other components. *O. myriophylla* (DY) and *O. hirta* (YM) are the most used *Oxytropis* varieties in Mongolian medicine. The two species have similar morphological characteristics but no specific chemical markers distinguishing them have been reported. The major bioactive components in the two species, based on HPLC-UV and HPLC-ELSD analysis were found to be saponins, flavonoids, and alkaloids (Okawa et al., 2002). However, a literature review (Baimukhambetov, 1973) observed that only a few flavonoids have been found in YM while several studies found that *Oxytropis racemosa* Turcz. (SZ) contained mostly flavonoids with few alkaloids (Song et al., 2010, 2013). In contrast, the major chemical components in *Oxytropis bicolor* Bge. (ES) were triterpenoids (Sun et al., 1991), with little evidence of flavonoids and alkaloids. This lack of information has significantly limited the clinical application of these medicinal plants.

Metabolomics is a powerful method that can detect global metabolite variations and discover specific markers in plant species. Metabolomic investigations are based on liquid chromatography-mass spectrometry (LC-MS), and in recent years, the use of ultra-high performance liquid chromatography-quadrupole time-of-flight mass spectrometry UHPLC-Q-TOF-MS has proved to be extremely useful for the rapid identification of metabolites in herbs due to its unsurpassed sensitivity and high resolution (Zhang et al., 2014; Yu et al., 2017; Zhao et al., 2018; Cui et al., 2020; Liu et al., 2020a). The use of UHPLC-Q-TOF-MS in metabolomics and in combination with different chemometric statistical tools is a versatile technique that can be effectively utilized to discover quality markers in the authentication of diverse herbal medicines (Masson et al., 2014; Wu et al., 2018; Liu et al., 2020b; Pan et al., 2020).

In this study, UHPLC-Q-TOF-MS was used as a rapid and accurate analytical method for the detection and characterization of the chemical constituents of *Oxytropis*. Principal component analysis (PCA) and orthogonal partial least squares discriminant analysis (OPLS-DA) were used to distinguish between the four *Oxytropis* species as well as between *O. myriophylla* obtained from different geographical regions based on specific markers. This method can accurately identify the different metabolites of the different plants, allowing a comprehensive analysis of their chemical components as well as the identification of specific marker compounds for distinguishing between the four species. The anti-inflammatory activity of the four *Oxytropis* species was analyzed *in vitro*. In combination with a review of the literature, this study investigated the chemical composition, pharmacological activities, and preliminary pharmacophylogeny of the species, proposing relationships between ethnopharmacology, pharmacology, and bioactive components. This study can provide a theoretical basis for a better understanding of *Oxytropis* and its utilization.

## Materials and methods

### Materials and reagents

The whole grass of *Oxytropis myriophylla* (Pall) DC. (DY), *Oxytropis hirta* Bunge. (YM), *Oxytropis racemosa* Turcz. (SZ), and *Oxytropis bicolor* Bge. (ES) were collected by Yang Liu from the Saihanwula Nature Reserve of Chifeng city in China (44°20′N, 118°30′E, elevation 1,440 m) on 12 August 2019. A further sample of *O.myriophylla* (DYWC) was collected in Wuchuan city, Inner Mongolia, China (41°16′N, 110°08′E, elevation 1,640 m) on 18 August 2019. Each *Oxytropis* contained six samples and were identified by associate professor Bi Qu of the college of pharmacy in Inner Mongolia medical university. The preserved leaf specimens are stored in the medicinal herbarium of the college of pharmacy at Inner Mongolia Medical University.

Reagents for metabolomic analysis included formic acid and methanol (LC-MS grade), purchased from Concord Technology (Tianjin, China) and acetonitrile from Sigma-Aldrich (St. Louis, MO, USA). Ultrapure water was used in all experiments.

For analyzing anti-inflammatory activity *in vitro*, RPMI 1,640 medium and Penicillin-Streptomycin (10,000 U/mL) were purchased from Gibco (USA), fetal bovine serum was purchased from ExCell Bio (Australia), NO kit was purchased from Nanjing Jiancheng (Nanjing, China), Mouse TNF-α ELISA KIT and Mouse IL-6 ELISA KIT purchased from Solarbio (Beijing, China). Mouse RAW 264.7 macrophages were obtained from the cell bank of the Chinese Academy of Sciences (Shanghai, China).

### Metabolomic analysis

Dried whole grass was ground to a fine powder and passed through a No. 100 mesh sieve. The powder sample (0.10 g) was dissolved in 5 mL of a 1:1 (v/v) methanol: water solution and soaked at room temperature for 12 h. The mixture was then ultrasonicated for 45 min and centrifuged (13,000 rpm, 10 min, 4°C) to obtain the supernatant.

The chromatographic separation was performed on an ExionLC system (AB Sciex, Foster City, CA, USA). A Waters Acquity BEH C_18_ column (2.1 × 100 mm, 1.7 μm) was used at a temperature of 35°C. The mobile phase consisted of 0.1% formic acid (A) and acetonitrile (B). The gradient conditions were: 0–2 min, 15 → 25% B; 2–6 min, 25 → 40% B; 6–9 min, 40 → 70% B; 9–11 min, 70 → 95% B; 11–13 min, 95% B; maintained at 15% B for an additional 10 min for re-equilibration.

For the high-resolution detection, a 5,600 Q-TOF mass spectrometer (AB Sciex) equipped with an electrospray ionization source (Turbo Ionspray) was used. MS detection was implemented in both the negative and positive ion modes. The analytical conditions used were as follows: gas1 and gas2, 55 psi; curtain gas, 35 psi. heat block temperature, 550°C; ion spray voltage, −4.5 kV in negative ion mode and 5.5 kV in positive ion mode, respectively; declustering potential, 50 V; collision energy, 40 V. QC samples were used to assess the system reproducibility and stability of the acquisition method by pooling small aliquots of each sample.

### Anti-inflammatory activity *in vitro*

For the extraction of total extracts, 10 g of dried medicinal powder from each of the four species was added to 150 mL of 70% ethanol solution. The solutions were extracted three times using heating and reflux (2 h per extraction). The extracts were then combined and concentrated under reduced pressure The total extracts of DY, YM, SZ, and ES were obtained as 1.7723, 1.7963, 1.4352, and 2.7994 g, and the extraction rates were 17.723, 17.963, 14.352, and 27.994%, respectively.

For cell culture and passaging, frozen RAW 264.7 cells were removed from liquid nitrogen and quickly thawed in a 37°C water bath. The supernatant was then removed by centrifugation, 1 mL of RPMI 1,640 was added to the complete medium containing 10% fetal bovine serum to resuspend the cells, after which the cell suspension was transferred to a Petri dish to be cultured in a incubator at 37°C, 5% CO_2_. The cell status was observed the next day. When the cells grew to 80–90% of the culture dish, they could be passaged. From the beginning of recovery, it is recorded as the first generation. Generally, cells from three to eight generations are selected for experiments. When the cell density was moderate, they were removed from the medium and fresh medium was added after centrifugation to make a cell suspension. The cells were then transferred to a Petri dish in a certain proportion to continue culturing in the incubator at 37°C, 5% CO_2_ for later use.

For MTT assays, cells in the logarithmic growth phase were selected, and the cell concentration was adjusted to 1 × 10^4^ cells/mL, then inoculated into a 96-well plate, 100 μL/well, and cultured in a 37°C, 5% CO_2_ incubator for 24 h. Different concentrations of the total extracts in the medium were added to cells in the experimental group, while equal volumes of the medium were added to cells in the control group. The cells were incubated with the extracts for 24 h. About 10 μL of MTT solution at 5 mg/mL was added per well and incubated in the dark for 4 h. The supernatant was then removed and 100 μL/well dimethyl sulfoxide (DMSO) was added and mixed evenly. After the purple crystal (formazan) was completely dissolved, the optical density (OD) of each well was measured using a microplate reader at the wavelength of 570 nm. The experiment was repeated three times to calculate the cell survival rate of each group.


(1) $$\begin{array}{l} \text{Cell Viability}=\;\left[\left(\text{As}-\text{Ab}\right)/\left(\text{Ac}-\text{Ab}\right)\right]\;\times 100 \end{array}$$

As: OD_570_ of experimental wells (medium containing cells, substance to be tested).
Ac: OD_570_ of control wells (medium containing cells, no substance to be tested).
Ab: OD_570_ of blank wells (medium without cells and substances to be tested).

For the establishment of the inflammatory cell model, RAW 264.7 cells in the logarithmic growth phase were selected, and the cell concentration was adjusted to 5 × 10^5^ cells/mL, and then inoculated in a 24-well plate, 500 μL/well, and cultured in a 37°C, 5% CO_2_ incubator for 24 h. The experimental group was incubated with different concentrations of lipopolysaccharide (LPS) (0.01, 0.1, 1, and 10 μg/mL), while the control group received only a culture medium. The NO concentrations were measured at 12, 24, and 48 h using a kit, according to the instructions. The experiment was repeated three times.

For the effect of LPS-induced RAW 264.7 cells on NO production and inflammatory factors, RAW 264.7 cells in the logarithmic growth phase were selected, and the cell concentration was adjusted to 5 × 10^5^ cells/mL, and then inoculated in a 24-well plate, 500 μL/well, and cultured in a 37°C, 5% CO_2_ incubator for 24 h. The cells were pretreated with different concentrations of total extracts for 1 h and then LPS was added for a total of 24 h. The positive drug group was added with LPS and indomethacin (INM, 100 μM). The model control group received only LPS, the blank control group received only the medium, and the NO concentrations were determined according to the instructions of the NO, TNF-α, and IL-6 kit. The experiment was repeated three times.

### Data processing and statistical analysis

The original obtained map was converted by XCMS. This included baseline filtering, peak identification, peak alignment, and other steps, and provided the data matrix, including the mass charge ratio (*m/z*), retention time (Rt), peak area (intensity), and other details. All data were normalized by the total peak area, and the generated Excel table was used for subsequent metabolome analysis. To reduce the signal interference caused by accidental error, the variables with RSD ≥ 40% in the quality control (QC) assessments were eliminated in Excel. The excel file was imported into SIMCA 14.1 (Umetrics, Umeå, Sweden) software for multivariate analysis, mainly PCA and OPLS-DA. The variable importance in projection (VIP) and S-plot were used to identify different components. The data of *in-vitro* anti-inflammatory activity experiments were statistically analyzed and plotted using GraphPad Prism 9.0 software. *T*-test was used for comparison between groups, and *P* < 0.05 was considered statistically different, *P* < 0.01 was considered statistically significantly different, and *P* < 0.001 was considered highly statistically different.

## Results

### Assignment of metabolic markers present in *Oxytropis* by UPLC-Q-TOF-MS

#### PCA analysis of four *Oxytropis*

**Figure 1:**
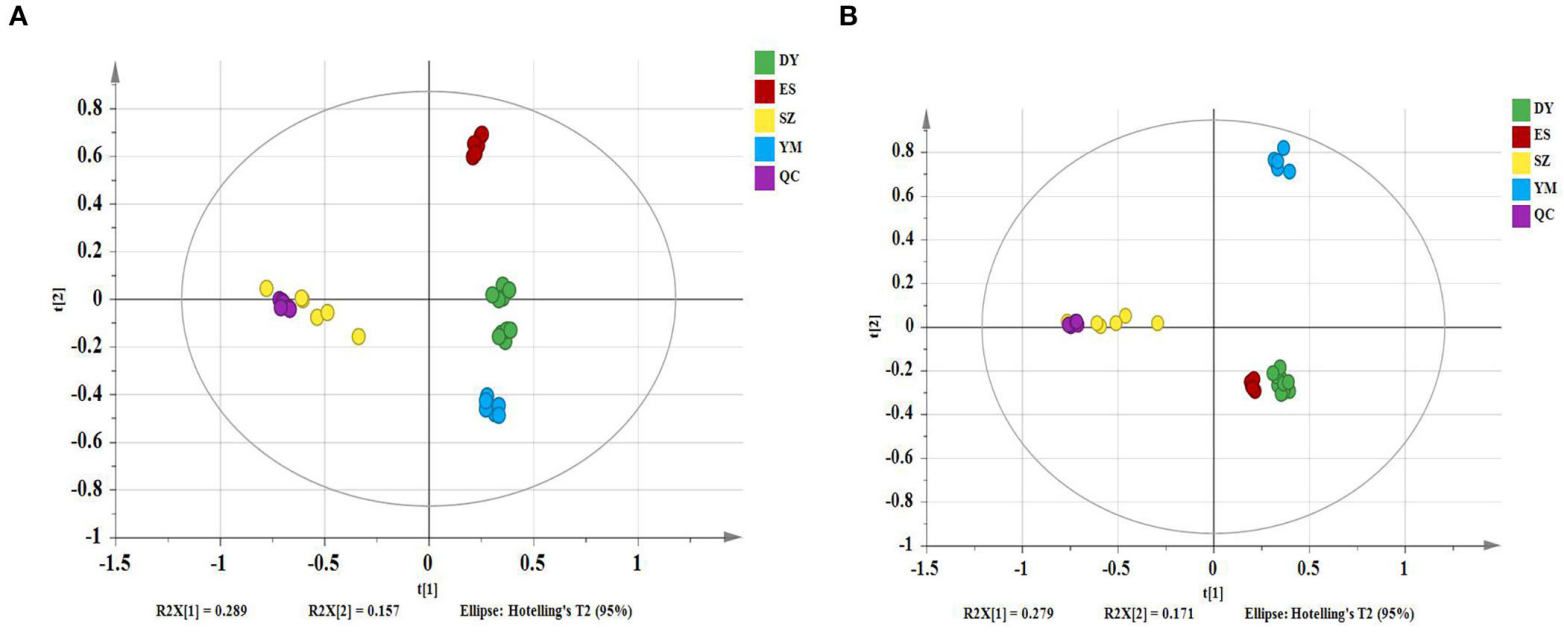
The principal component analysis (PCA) scatter plot of different *Oxytropis* species [**(A)** positive mode; **(B)** negative mode].

The PCA models containing all samples were first established to assess the distribution of the QC samples and the distances between the four species. The model showed 28.9% of variations in X (R2X[1] = 0.289), 15.7% of variations in response X (class) (R2X[2] = 0.157), and 60.3% of variations in response Y (Q2Y = 0.603) in the positive mode (Figure 1A). The model described 27.9% of the variations in X (R2X[1] = 0.279), 17.1% of the variations in response X (class)(R2X[2] = 0.171), and 62.2% of the variations in response Y (Q2Y = 0.622) in the negative mode (Figure 1B). It can be seen from the figures that under the positive and negative ion modes, the QC samples are closely gathered together, confirming both the stability and repeatability of the experiment and that the data were stable, reliable, and effective. In addition, it was clear that the different *Oxytropis* samples could be distinguished, indicating significant differences in the chemical components of the different *Oxytropis* varieties.

#### Global identification of chemical components in four *Oxytropis*

**Figure 2:**
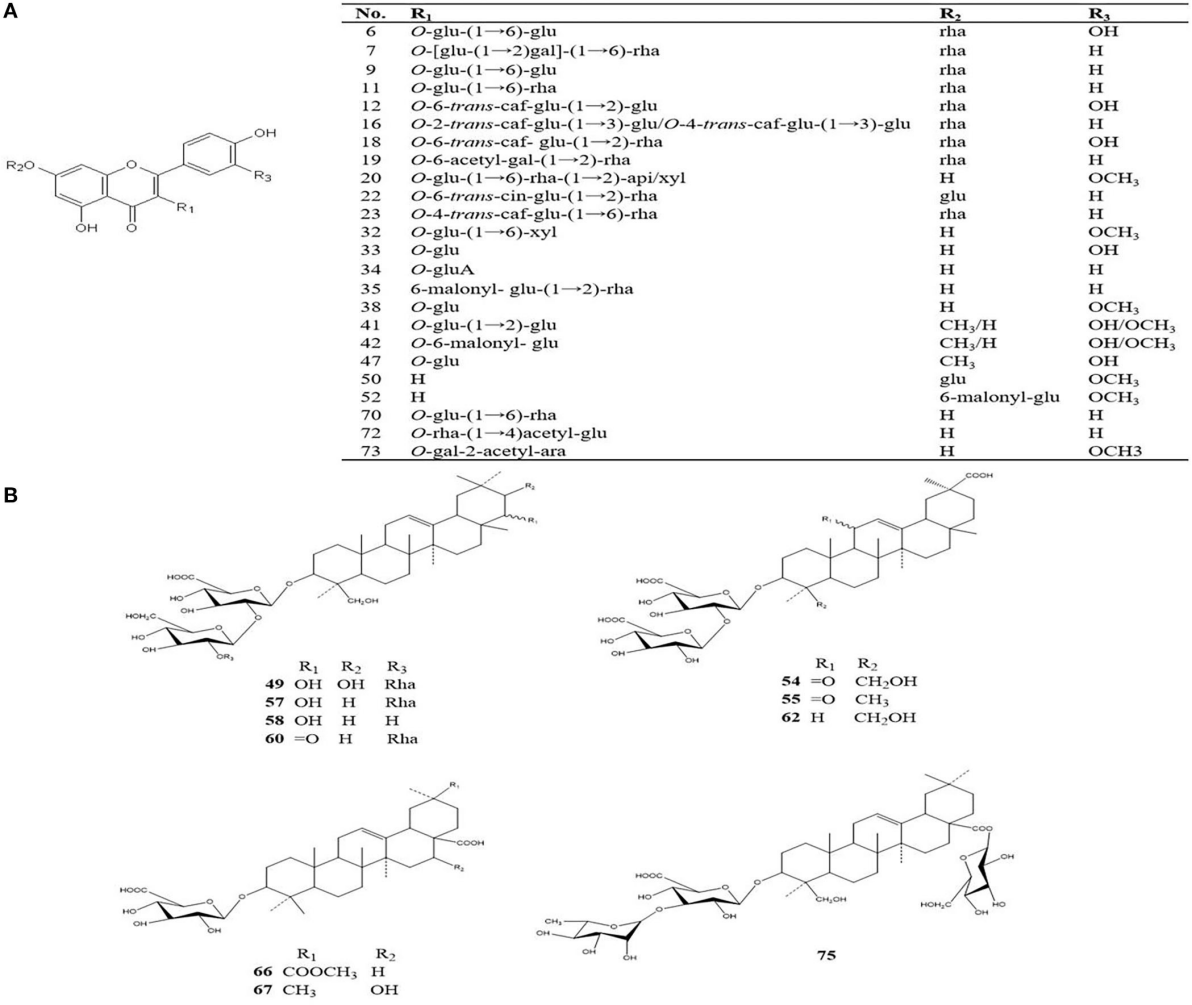
Typical chemical structures of flavonoid glycosides and saponins identified from *Oxytropis* [**(A)** flavonoids; **(B)** saponins].

The four different varieties of *Oxytropis* were globally characterized by UHPLC-QTOF-MS. As the differences between the four species could be directly observed from base peak chromatography (BPC), Supplementary Figure S1 shows the positive and negative ion modes in the BPC diagram of the *Oxytropis* samples. Additionally, using the information from MS/MS chromatogram together with information retrieved from the literature (Sun and Chen, 1997; Song et al., 2010, 2013; Li et al., 2012; Masafumi et al., 2022) and public databases, a total of 75 compounds were identified (Supplementary Table S1). Compounds in each *Oxytropis* species were classified by their retention times, fragment ions, accurate molecular mass, and credible molecular formulas and chemical names. Compounds belonging to various classes were identified in the four species, including 42 flavonoid glycosides, 14 saponins, 5 alkaloids, 5 amino acids, 3 organic acids, and 6 other compounds. Figure 2 shows the typical chemical structures of the flavonoid glycosides and saponins identified in *Oxytropis*. The flavonoid glycosides and saponins formed the two largest categories among the 75 identified compounds.

**Figure 3:**
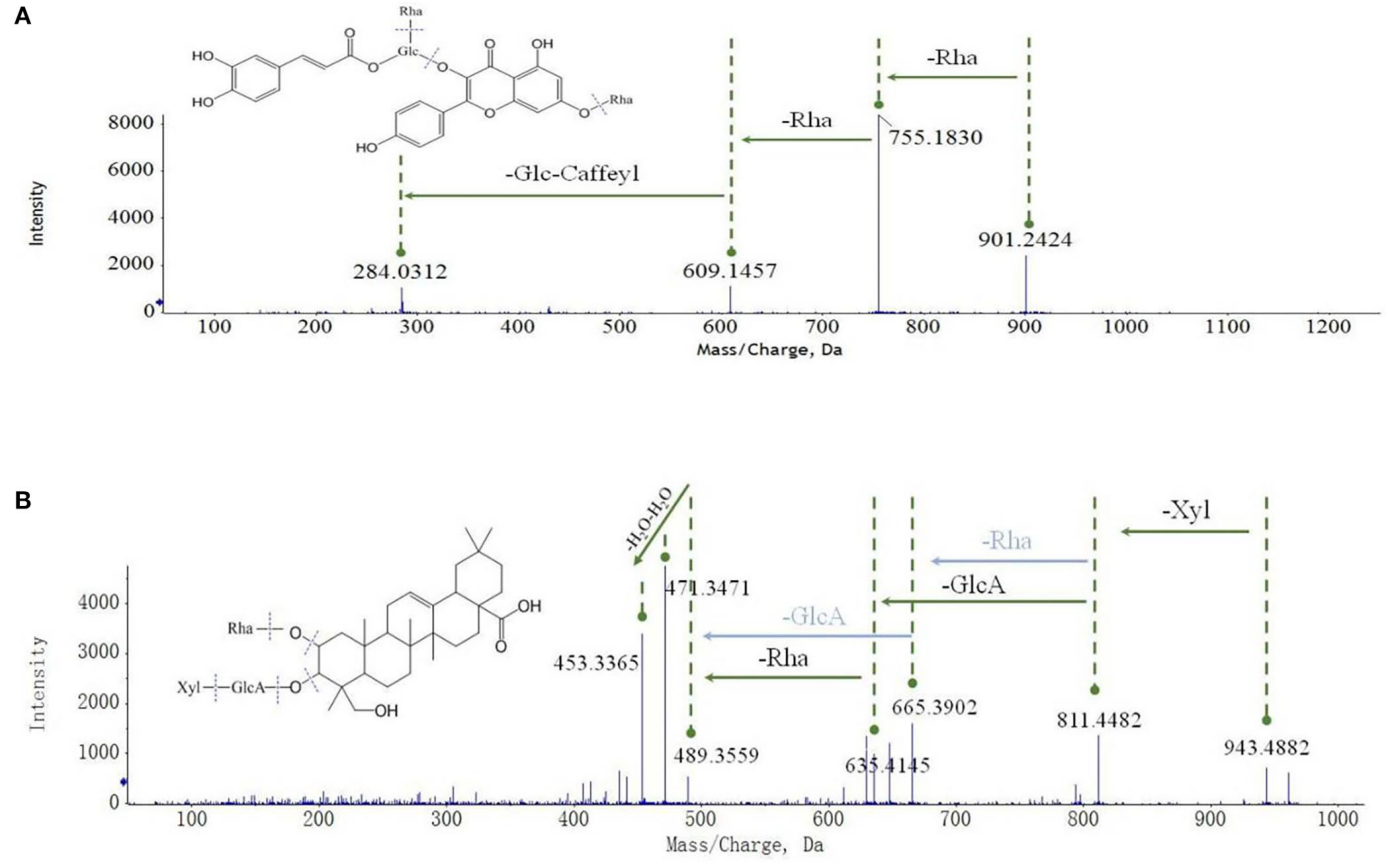
Mass spectrum cleavage law of flavonoids and saponins [**(A)** flavonoids; **(B)** saponins].

*Oxytropis myriophylla* (DY) is the most commonly used species in Mongolian medicine, and it contained compounds present in all four *Oxytropis* varieties**. **In all, 43 compounds were detected in DY, including 21 flavonoid glycosides, 6 saponins, 3 alkaloids, and 5 amino acids, among other compounds (Supplementary Table S1). The majority of these compounds have been reported to have significant biological activities. For instance, the flavonoid glycoside kaempferol 3-caffeylrobinobioside-7-rhamnoside (Compound 23) was only detected in DY. Figure 3A shows the MS/MS mass spectrum of compound 23. The precursor ion [M–H]^−^ was at *m/z* 901.2424 found at 3.258 min. The compound formula was predicted to be C_42_H_46_O_22_. The different daughter ions at *m/z* 755.1830, 609.1457, and 284.0312 were [M–H–Rha]^−^, [M–H–Rha–Rha],^−^, and [M–H–Rha–Rha–Glc–Caffeyl]^−^, respectively.

*Oxytropis hirta* (YM) is also a commonly used *Oxytropis* species in Mongolian medicine, and to date, few compounds have been isolated from YM. In this study, ~30 different compounds were detected in YM. These included 3 alkaloids and 8 saponins as well as other compounds including flavonoid glycosides. Three saponins, namely, licoricesaponin G2, licoricesaponin J2, and glycyrrhizic acid were only found in YM (Supplementary Table S1). Recent studies have reported that licorice, which is a triterpene saponin, shows significant anti-inflammatory, anticancer, and hepatoprotective activities (Tang et al., 2015; Zheng et al., 2015; Yang et al., 2017).

In *Oxytropis racemosa* (SZ), we identified 10 saponins besides flavonoid glycosides and alkaloids that have been previously reported. For instance, triterpenoid-xyl-rha-gluA (Compound 51) was only detected in SZ. The general fragmentation pathways are illustrated in Figure 3B. The precursor ion [M + H]^+^ *m/z* 943.4882 was observed at 6.831 min, while the other fragments were seen at *m/z* 811.4482, 635.4145, 489.3559, 471.3471, and 453.3365. Interestingly, the presence of *m/z* 811.4482 fragments indicated the loss of xylose from the precursor ion, MS/MS fragments *m/z* 635.4145 suggested the loss of glucuronic acid from the product ion at *m/z* 811.4482, and *m/z* 489.3559 indicated a loss of rhamnose monohydrate from the product ion at *m/z* 635.4145, whereas *m/z* 453.3365 indicated loss of H_2_O from the product ion at *m/z* 471.3471.

Thirty compounds were detected in *Oxytropis bicolor* (ES), principally 9 flavonoid glycosides, 8 saponins, 2 alkaloids, and other compounds. Of these, 6 flavonoid glycosides were detected only in ES (Supplementary Table S1). These results may provide a meaningful basis to develop and promote the pharmacological use of ES in the future.

#### Visualization of the distribution of marker compounds by heatmap

**Figure 4:**
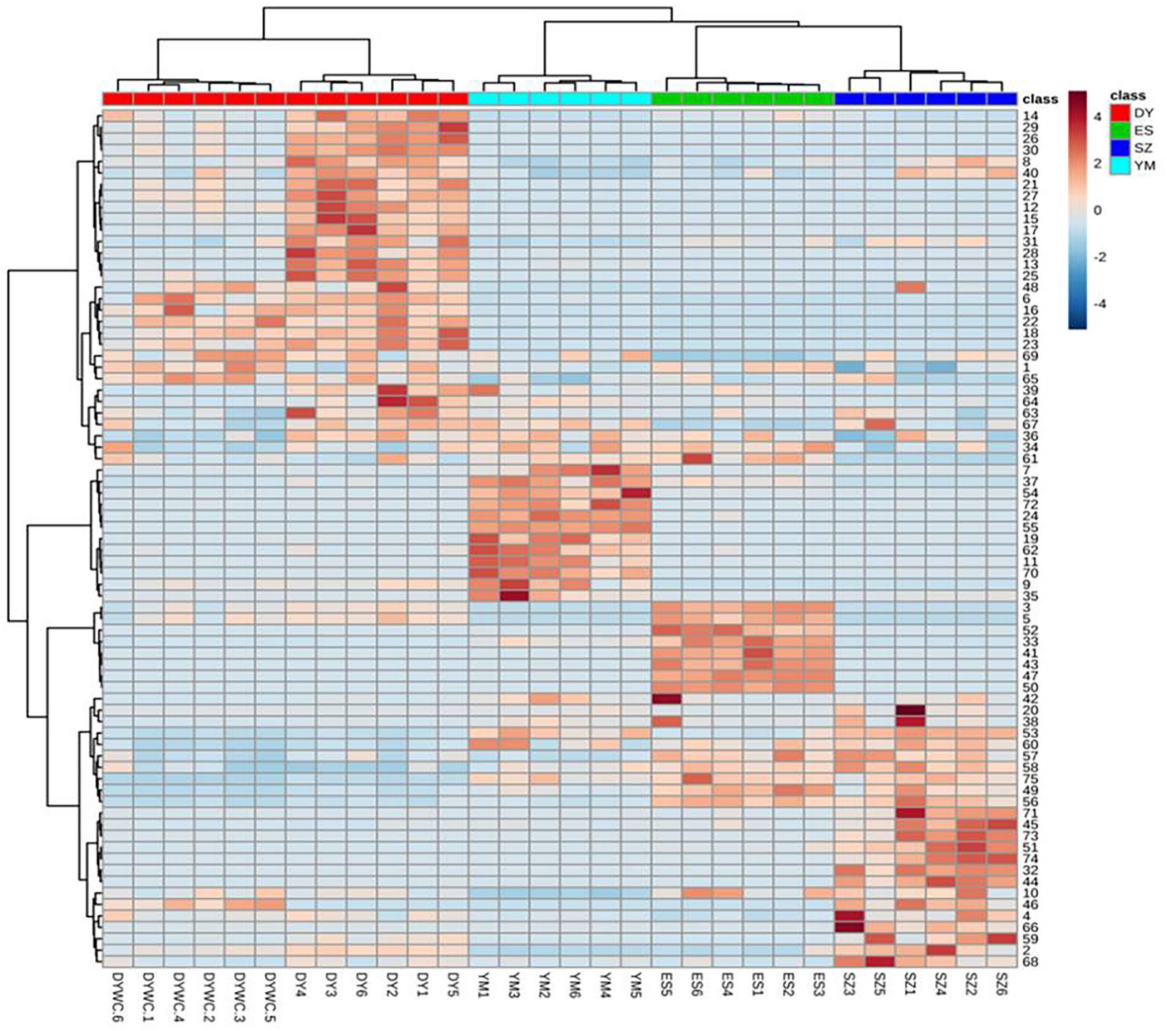
The heat map of identified differential metabolites of *Oxytropis*. (Darker brown indicates higher contents. The figures on the right correspond to compound number order in Supplementary Table S1).

A heatmap visualization was generated to provide detailed information on the distribution of the marker compounds present in *Oxytropis*. As shown in Figure 4, the four *Oxytropis* species were clearly classified with the “unsupervised” clustering analysis. The samples were further classified automatically. The results indicated that the identified 75 components served as representatives in classifying *Oxytropis*. In the heatmap, each cell represents an individual compound, with darker colors indicating higher contents. Each sample shows a dark color block, indicating the specific compounds present in the different *Oxytropis* species. For example, quercetin3-*O*-gentiobioside-7-*O*-rha, sarmenoside III, and kaempferol 3-caffeylrobinobioside-7-rhamnoside were highly enriched in DY, while licoricesaponin G2, glycyrrhizic acid, and licoricesaponin J2 were significantly enriched in YM (Figure 4). DY and ES contained the highest proportions of the 75 identified compounds and the compound proportion was higher in DY than in DYWC. According to the distribution of each component in the heatmap, 23 potential marker compounds (Table 2) were identified to establish a Support Vector Machine (SVM) model for classification and prediction. In the model, black is the classification result (training set) of modeling, and red is the prediction result (test set) after modeling. The classification accuracy is 100%. The two results overlap, indicating that the model has good prediction ability and can be used for the identification of different basic sources of *Oxytropis*. The results are shown in Supplementary Figure S2.

**Table 2:** Components for SVM establishment.

**No**.	**Identification**	**Formula**	**Class**	**Source**
6	Quercetin 3-*O*-gentiobioside-7-*O*-rha	C_33_H_40_O_21_	Flavonoid glycoside	DY
16	Kaempferol 3-(2”-caffeyllaminaribioside)-7-rhamnoside/kaempferol 3-(4”-caffeyllaminaribioside)-7-rhamnoside	C_42_H_46_O_23_	Flavonoid glycoside	DY
18	Sarmenoside III	C_42_H_46_O_23_	Flavonoid glycoside	DY
22	3-(β-D-Glucopyranosyloxy)-4',5-dihydroxy-7-[2-*O*-[6-*O*-(3-methoxy-4-hydroxy-trans-cinnamoyl)-β-D-glu]-α-L-rhamnopyranosyloxy]flavone	C_43_H_48_O_23_	Flavonoid glycoside	DY
23	Kaempferol 3-caffeylrobinobioside-7-rhamnoside	C_42_H_46_O_22_	Flavonoid glycoside	DY
41	Rhamnetin 3-sophoroside/isorhamnetin 3-laminaribioside/rhamnetin 3-laminaribioside	C_28_H_32_O_17_	Flavonoid glycoside	ES
43	Chrysoeriol-7-*O*-(2”-*O*-mannopyranosyl)allopyranoside/complanatuside	C_28_H_32_O_16_	Flavonoid glycoside	ES
45	3-[6-*O*-[4-*O*-(4-Oxo-4-hydroxybutyryl)-6-deoxy-α-L-mannopyranosyl]-β-D-glu]-3',4',5,7-tetrahydroxyflavone	C_31_H_34_O_19_	Flavonoid glycoside	ES
47	Rhamnetin 3-*O-β*-glucopyranoside	C_22_H_22_O_12_	Flavonoid glycoside	ES
50	Thermopsoside/kaempferide 7-glucoside	C_22_H_22_O_11_	Flavonoid glycoside	ES
52	Luteolin 3'-methyl ether 7-malonylglucoside/quercetin 3-(3”,6”-diacetylgalactoside)	C_25_H_24_O_14_	Flavonoid glycoside	ES
32	Isorhamnetin 3-*O*-[β-D-xylopyranosyl-(1 → 6)-β-D-glucopyranoside]	C_27_H_30_O_16_	Flavonoid glycoside	SZ
44	[(2R,3R)-2-(3,4-dihydroxyphenyl)-5,7-dihydroxy-3,4-dihydro-2H-chromen-3-yl]oxymethyl hydrogen carbonate	C_17_H_16_O_9_	Flavonoid glycoside	SZ
51	Triterpenoid-xyl-rha-gluA	C_47_H_74_O_19_	Saponin	SZ
71	Herbacetin 7-(6”-quinoylglucoside)	C_28_H_30_O_17_	Flavonoid glycoside	SZ
73	Isorhamnetin 3-(2”'-acetyl-α-arabinopyranosyl)-(1 → 6)-galactoside/tricetin 4'-methyl ether 7-apiosyl-(1 → 2)-(6”-acetylglucoside)	C_29_H_32_O_17_	Flavonoid glycoside	SZ
74	Unknown	C_31_H_34_O_18_	Flavonoid glycoside	SZ
19	Kaempferol 3-(2”-rhamnosyl-6”-acetylgalactoside) 7-rha	C_35_H_42_O_20_	Flavonoid glycoside	YM
24	Unknown	C_15_H_19_NO_8_	Glucoside	YM
54	Licoricesaponin G2	C_42_H_62_O_17_	Saponin	YM
55	Glycyrrhizic acid	C_42_H_62_O_16_	Saponin	YM
62	Licoricesaponin J2	C_42_H_64_O_16_	Saponin	YM
72	3-[4-*O*-(6-*O*-acetyl-β-D-glu)- α-L-rhamnopyranosyloxy]-4',5,7-trihydroxyflavone/kaempferol 3-*O*-(6”-*O*-acetyl)glucoside-7-*O*-rhamnoside/multiflorin A	C_29_H_32_O_16_	Flavonoid glycoside	YM

As reported earlier, flavonoid glycosides and saponins were found to be the main bioactive compounds in *Oxytropis*. Moreover, in our study, the relative concentrations of some flavonoid glycoside and saponin compounds were different in DY plants from different geographical origins. Therefore, further studies are required to understand the basis of the difference.

#### Comparison of marker compounds in*O. myriophylla* obtained from different geographical regions

**Figure 5:**
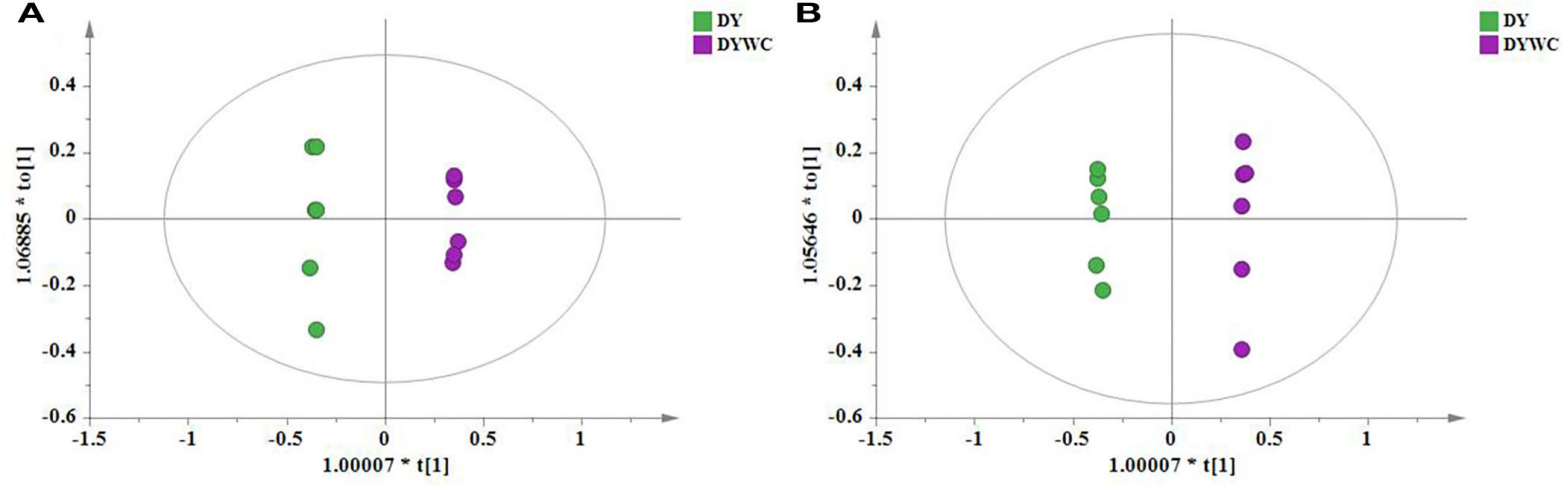
OPLS-DA of DY and DYWC [**(A)** positive mode; **(B)** negative mode].

To identify potential marker compounds and distinguish between *O. myriophylla* plants from different geographical regions, a supervised OPLS-DA was conducted. The grouping variable area was adopted with pair-wise analysis which can make the differences clearer and more straightforward. OPLS-DA has been regularly used to screen different metabolites. Figure 5 shows the OPLS-DA of DY and DYWC in both the positive and negative modes, indicating that the two groups could be clearly distinguished.

**Figure 6:**
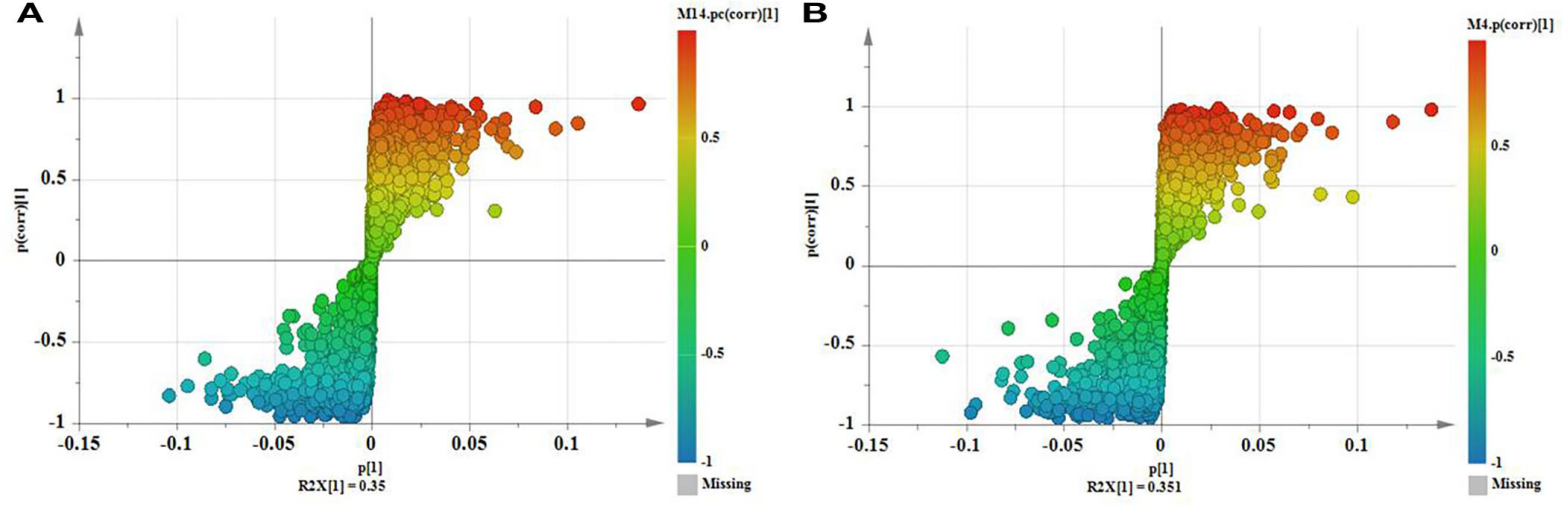
S-Plot of DY and DYWC [**(A)** positive mode; **(B)** negative mode].

Variables with VIPs > 1 in the model were considered to contribute significantly to differences between the plants. Partial correlation coefficients (Pcorrs) were also used to identify the most influential and important variables. While the Pcorr value itself does not indicate any uniform screening principles, higher Pcorr values indicate greater contributions to the model. In this experiment, Pcorr > 0.5 was set as the screening criterion. As seen in Figure 6, the S-plot can also visually indicate the exact contribution of each variable. The points further from the origin of the e-coordinates in the “S” segment clearly showed that the compounds from different geographical origins were significantly different (Li et al., 2018).

After screening variables using VIP and Pcorr, the Mann-Whitney *U* test was used to calculate the *p*-values. A total of 26 potential marker compounds were identified in the two DY plant samples from different geographical regions using the *S* scatter plot. The results are shown in Supplementary Table S2. Interestingly, while no major differences in the types of compounds identified were observed between DY and DYWC, significant differences in their concentrations were found with higher concentrations apparent in DY plants from Saihanwula. For example, the concentrations of several prominent amino acids such as DL-phenylalanine and proline were almost 0.7-fold higher in DY compared with DYWC. Proline is widely considered to play a key role in maintaining osmotic balance and thus has an important osmoprotective function in plant defense mechanisms. As an osmotic agent, the different concentrations of this amino acid in plants from different regions suggest a reflection of different environmental conditions between the areas (Szabados and Savoure, 2010; Sharma et al., 2011). Aromatic amino acids, such as phenylalanine, serve as precursors for the biosynthesis of polyphenols and flavonoids. This can possibly explain the reason for the higher concentrations of most components seen in DY plants from Saihanwula compared with Wuchuan. For instance, 13 flavonoid glycoside compounds were found to be present in higher concentrations in DY from Saihanwula with kaempferol 3-caffeylrobinobioside-7-rhamnoside and additional unknown compounds serving as representatives. Additionally, few saponins, alkaloids, and other compounds were found. Similarly, the SVM model was established for the classification and prediction of DY plants from the two different geographical regions. The results showed a 100% classification accuracy, and the predicted results can overlap with the actual results, thereby indicating that the model has good predictive power (Supplementary Figure S3). This model can potentially be used to distinguish between DY plants from different geographical regions.

### Anti-inflammatory activity of *Oxytropis* extracts

#### Effects of extracts on cell proliferation

**Figure 7:**
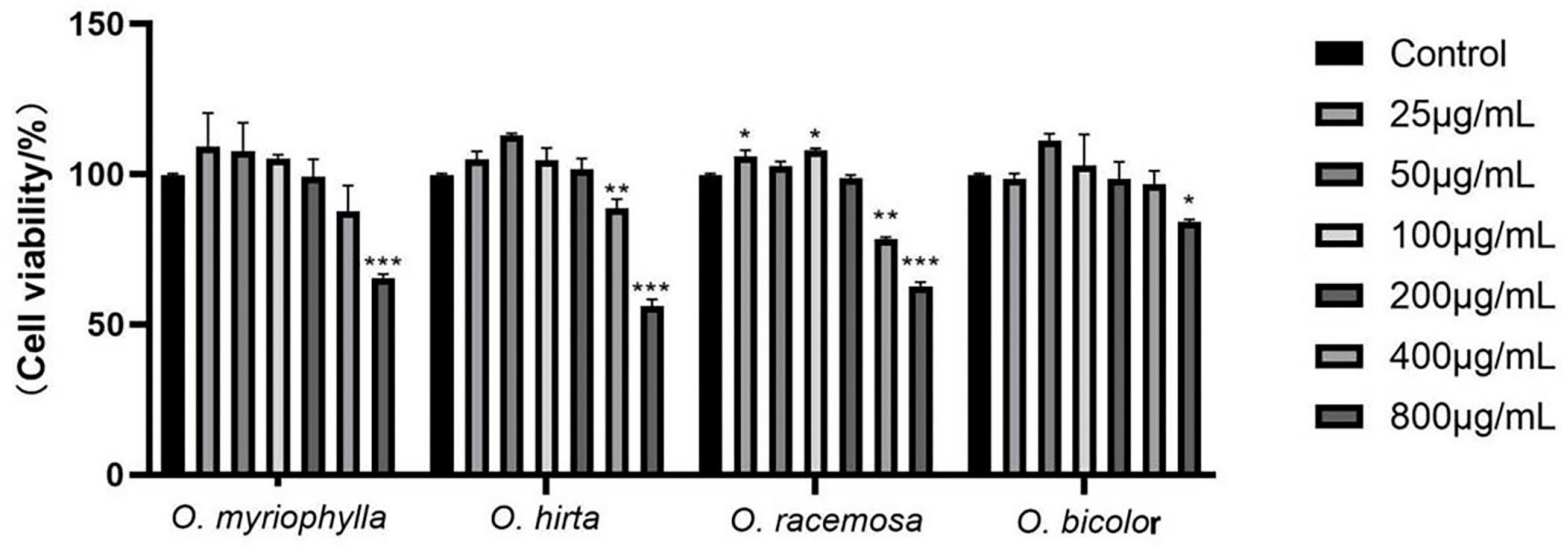
Effects of the total extracts of the four *Oxytropis* on the proliferation of mouse RAW264.7 macrophages (Compared with the control group, *P < 0.05, **P < 0.01, and ***P < 0.001).

All four *Oxytropis* extracts were non-cytotoxic to RAW 264.7 cells at mass concentrations up to 200 μg/mL, and there was no significant difference compared with the control group. Therefore, the extract concentrations of 12.5, 50, and 200 μg/mL (low-, medium-, and high-dose groups) were selected for subsequent experiments. The MTT experimental results of the four *Oxytropis* extracts are shown in Figure 7.

#### Establishment of LPS-induced RAW 264.7 macrophage inflammation model in mouse

**Figure 8:**
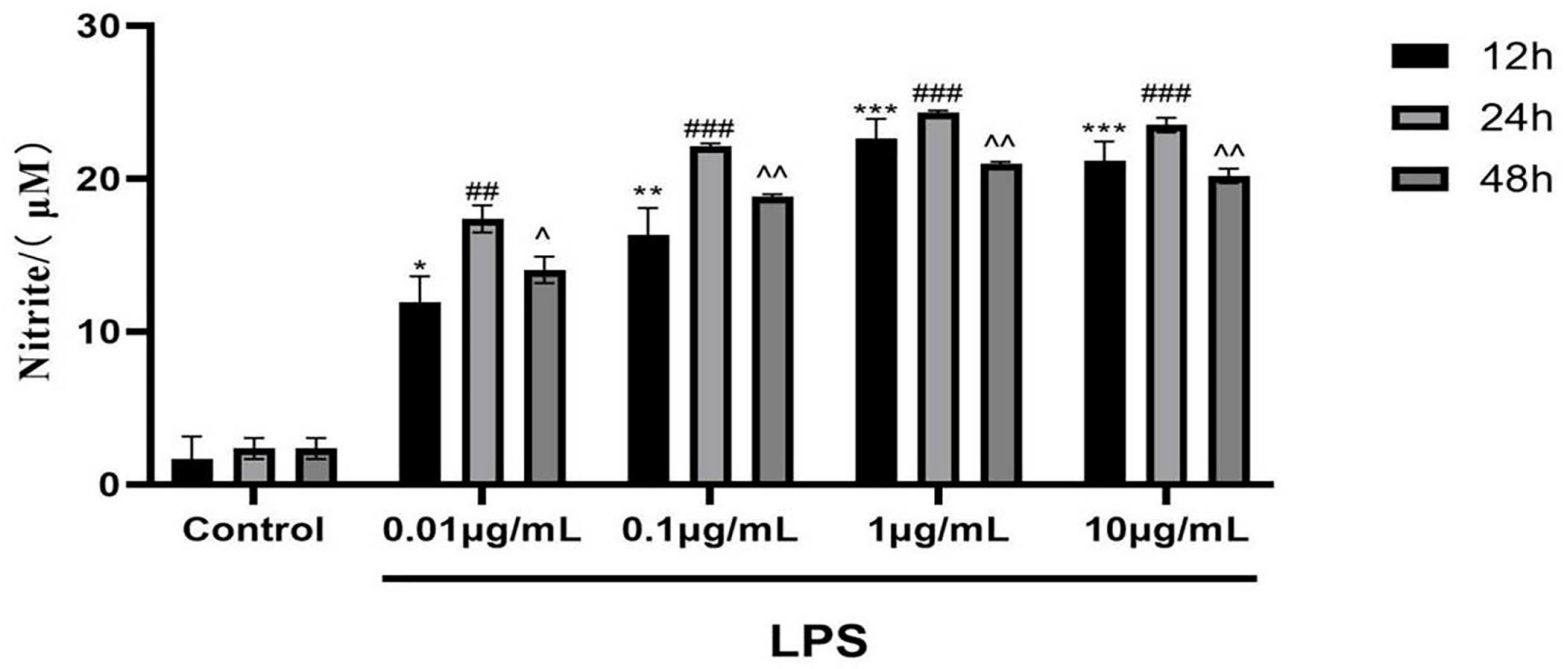
Effects of different concentrations of LPS for different times on NO production in mouse RAW 264.7 macrophages (Compared with the control group, at 12, 24 and 48 h respectively, *P < 0.05, **P < 0.01, ***P < 0.001; ^##^*P* < 0.01, ^###^*P* < 0.001; ^∧^*P* < 0.05, ^∧∧^*P* < 0.01).

LPS concentrations of 0.01, 0.1, 1, and 10 μg/mL were used. The NO production in RAW 264.7 cells at 12, 24, and 48 h is shown in Figure 8. When the LPS concentration was 1 μg/mL for 24 h, NO production by the cells was highest, showing a highly significant difference in comparison with the blank control group (*P* < 0.001). These results indicate the successful establishment of the LPS-induced RAW 264.7 macrophage inflammation model. Therefore, follow-up experiments were carried out with an LPS concentration of 1 μg/mL and an incubation time of 24 h as the optimal modeling conditions.

#### Effects of LPS-induced NO production in mouse RAW 264.7 macrophages

**Figure 9:**
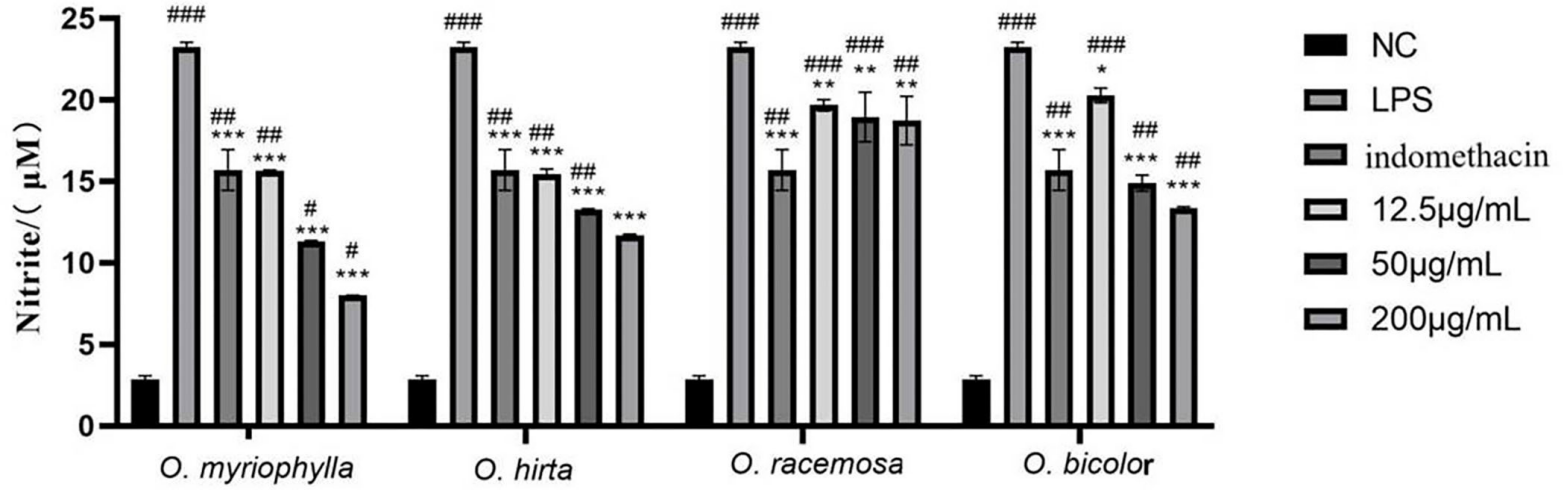
Effects of the total extracts of the four *Oxytropis* on NO production in LPS-induced mouse RAW 264.7 macrophages (NC, blank control group; LPS, 1 μg/mL LPS group; indomethacin: 100 μM indomethacin + 1 μg/mL LPS group. Compared with the NC group, ^#^*P* < 0.05,^##^*P* < 0.01,^###^*P* < 0.001;compared with the model group (LPS group),*P < 0.05, **P < 0.01,***P < 0.001).

**Figure 10:**
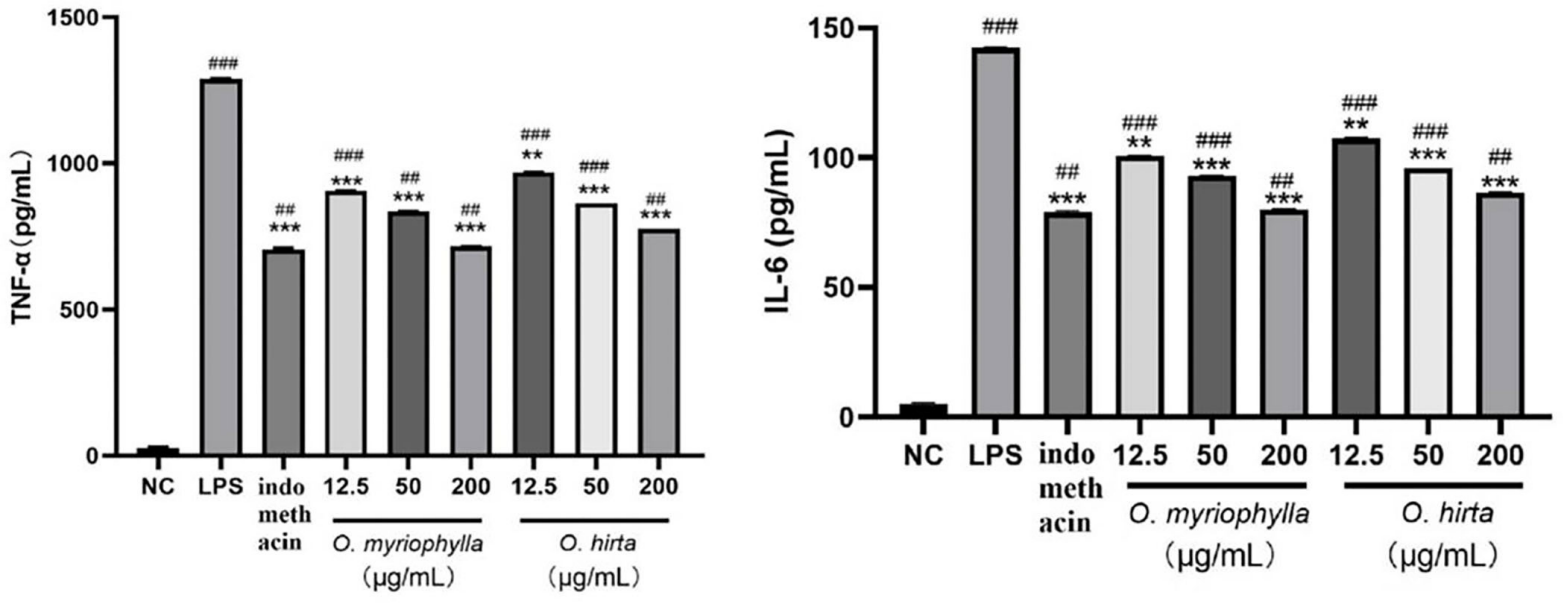
Effects of *O. myriophylla* and *O. hirta* on LPS-induced TNF-α and IL-6 expression in mouse RAW 264.7 macrophages (NC, blank control group; LPS, 1 μg/mL LPS group; indomethacin: 100 μM indomethacin + 1 μg/mL LPS group. Compared with the NC group, ^##^*P* < 0.01, ^###^*P* < 0.001; compared with the model group (LPS group), **P < 0.01, ***P < 0.001).

The effects of the four total extracts on LPS-induced NO production in RAW 264.7 cells are shown in Figure 9. The NO levels in the cultured supernatants of each group were significantly higher than those in the blank control group, indicating the successful establishment of the RAW 264.7 macrophage inflammation model. At low, medium, and high doses, the different total extracts could significantly reduce NO levels in the cultured supernatants (*P* < 0.001, *P* < 0.01, or *P* < 0.05), and had different anti-inflammatory effects. Specifically, *O. myriophylla* and *O. hirta* reduced NO production to a greater extent compared to the LPS group, exhibited stronger anti-inflammatory activity, and the effects of *O. myriophylla* and *O. hirta* on inflammatory factor TNF-α and IL-6 expression were examined (Figure 10). The inhibition rates of NO secretion at low, medium, and high doses are shown in Table 3.

**Table 3:** NO inhibition rate of total extracts from *Oxytropis*.

**Total extracts**	**NO inhibition rate (%)**		
	**Low dose**	**Medium dose**	**High dose**
*O. myriophylla*	33.51	51.30	65.54
*O. hirta*	32.74	42.92	49.64
*O. bicolor*	12.79	35.90	42.51
*O. racemosa*	15.36	18.45	19.43

## Discussion

### Characteristic marker components of *Oxytropis*

At present, with the shortage of wild resources, more *Oxytropis* species are currently being used in clinical applications. The analysis of overall chemical composition is thus of great significance to compare the differences between *Oxytropis* varieties. However, to date, only one or a few indices have been used to assess the compositions of the plant extracts, either qualitatively or quantitatively, which cannot fully reflect the quality. Our preliminary findings on the chemical constituents of *Oxytropis* using traditional chemical methods only identified a few compounds. Further studies were thus needed. In this study, we conducted a metabolomics investigation using UHPLC–Q-TOF–MS to characterize the compositions of four different *Oxytropis* species and assessing the differences between *O. myriophylla* from two different geographical regions. Metabolomics is a highly sensitive high-throughput technique that is widely used in medical research (Yao et al., 2019; Xia et al., 2021). However, there have been no reports on *Oxytrop*is quality control and evaluation with metabolomics. In the present study, we used positive and negative ion analysis to study the overall chemical composition of the *Oxytropis* species. This, combined with multivariate statistical analysis, was able to identify the different chemical components of the plant species.

The metabolites of medicinal plants have different types and structures. They often vary significantly according to time and space (Anne et al., 2020) and are influenced by different environmental conditions. The Saihanwula Nature Reserve of Chifeng city in China and Wuchuan city in Inner Mongolia of China are the main areas where *O. myriophylla* grows. We compared the chemical compositions and analyzed the relative contents of differential metabolites from the two different geographical origins with multivariate statistical analysis. Although no significant differences in the types of metabolites were found between DY and DYWC, there were significant differences in their concentrations. Most of the components showed higher concentrations in DY from the Saihanwula Nature Reserve of Chifeng city. The metabolic pathways and bioactive compounds present in the plants can be significantly affected by different environmental conditions (Ballhorn et al., 2011). For instance, different environmental conditions, such as variations in light, temperature, pH, and soil conditions, can all lead to substantial alterations in the metabolic pathways and the subsequent accumulation of secondary metabolites (Ramakrishna and Ravishankar, 2014; Jia et al., 2015). Saihanwula Nature Reserve and Wuchuan city are geographically distant. The significant differences in metabolite concentrations between DY plants from Saihanwula and Wuchuan may thus be attributed to the different environmental conditions in these two regions. Furthermore, these variations may also lead to marked differences in both the quality and pharmacological activities of the plant extracts.

### Preliminary pharmacophylogenetic investigation in *Oxytropis*

**Figure 11:**
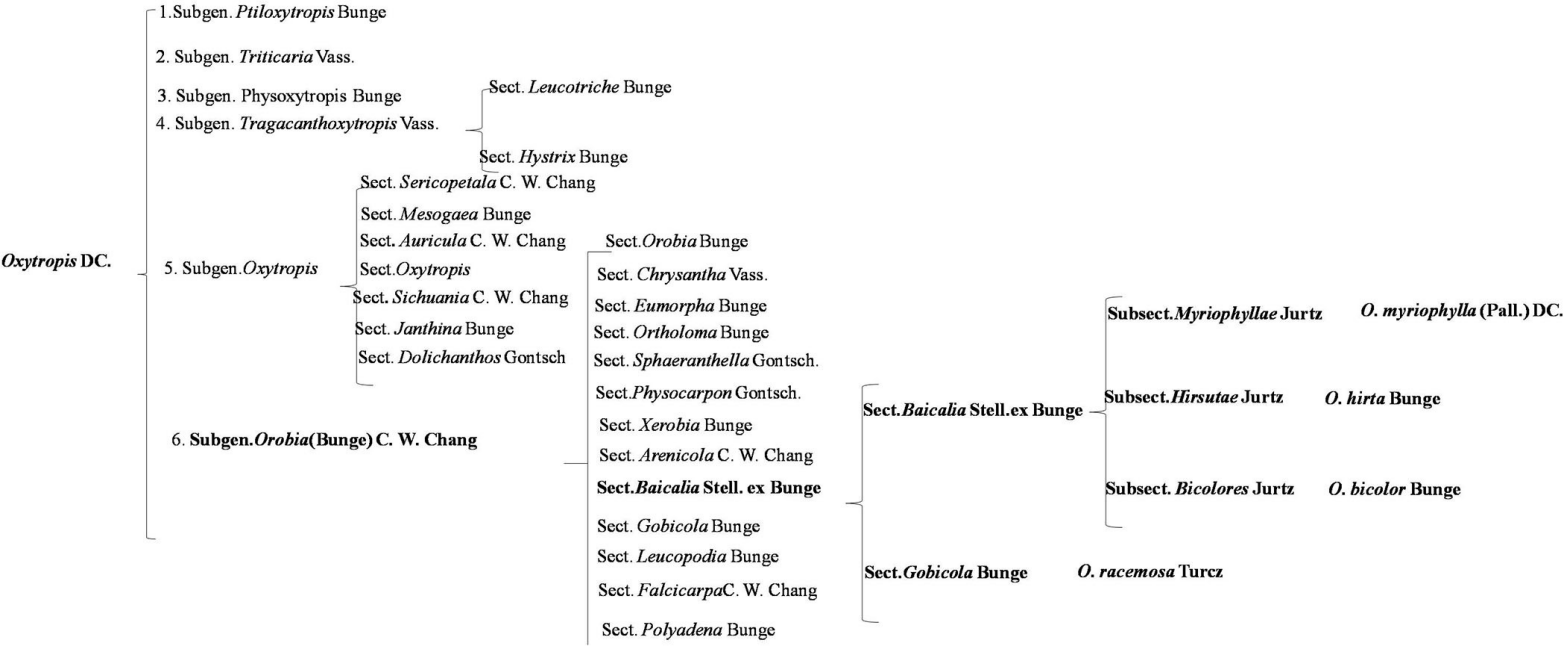
Plant chemotaxonomy of the four *Oxytropis*.

*Oxytropis* is usually divided into six subgenera (Figure 11). They are Subgen. *Ptiloxytropis* Bunge, Subgen. *Triticaria* Vass, Subgen. *Physoxytropis* Bunge, Subgen. *Tragacanth oxytropis* Vass., Subgen. *Oxytropis*, and Subgen. *Orobia* (Bunge) C. W. Chang. The four *Oxytropis* species investigated in this study belong to the Subgen. *Orobia* (Bunge) C. W. Chang, which contains 13 Sect. *Oxytropis myriophylla, Oxytropis hirta*, and *Oxytropis bicolor* belong to Sect. *Baicalia* Stell. ex Bunge, while *Oxytropis racemosa* belongs to Sect. *Gobicola* Bunge. From the perspective of plant taxonomy, *O. myriophylla, O. hirta*, and *O. bicolor* are more closely related.

Flavones are the major components of this genus. From the isolated flavonoids in these four *Oxytropis* mainly exist in the form of flavonoid glycosides. The aglycons mainly include quercetin, kaempferol, and isorhamnetin, most of which are flavones. The sugar substituents are mainly glucose and rhamnose, in addition to galactose, xylose, arabinose, and mannose. The results are in agreement with the findings of Li et al. (2012) and Song et al. (2013). Although the structure of flavonoid glycosides is relatively simple in the four *Oxytropis*, there is one distinguishing feature; in all oxyglycosides, the glycosidic bonds were mostly in positions three and seven.

Besides flavonoids, triterpenoids exist in the form of triterpenoid saponins in the four *Oxytropis*, most of which are oleanane type with 3-*O* substitution, and the sugar substituents are glucose and rhamnose. Alkaloids are mainly indole alkaloids in the four *Oxytropis*.

**Figure 12:**
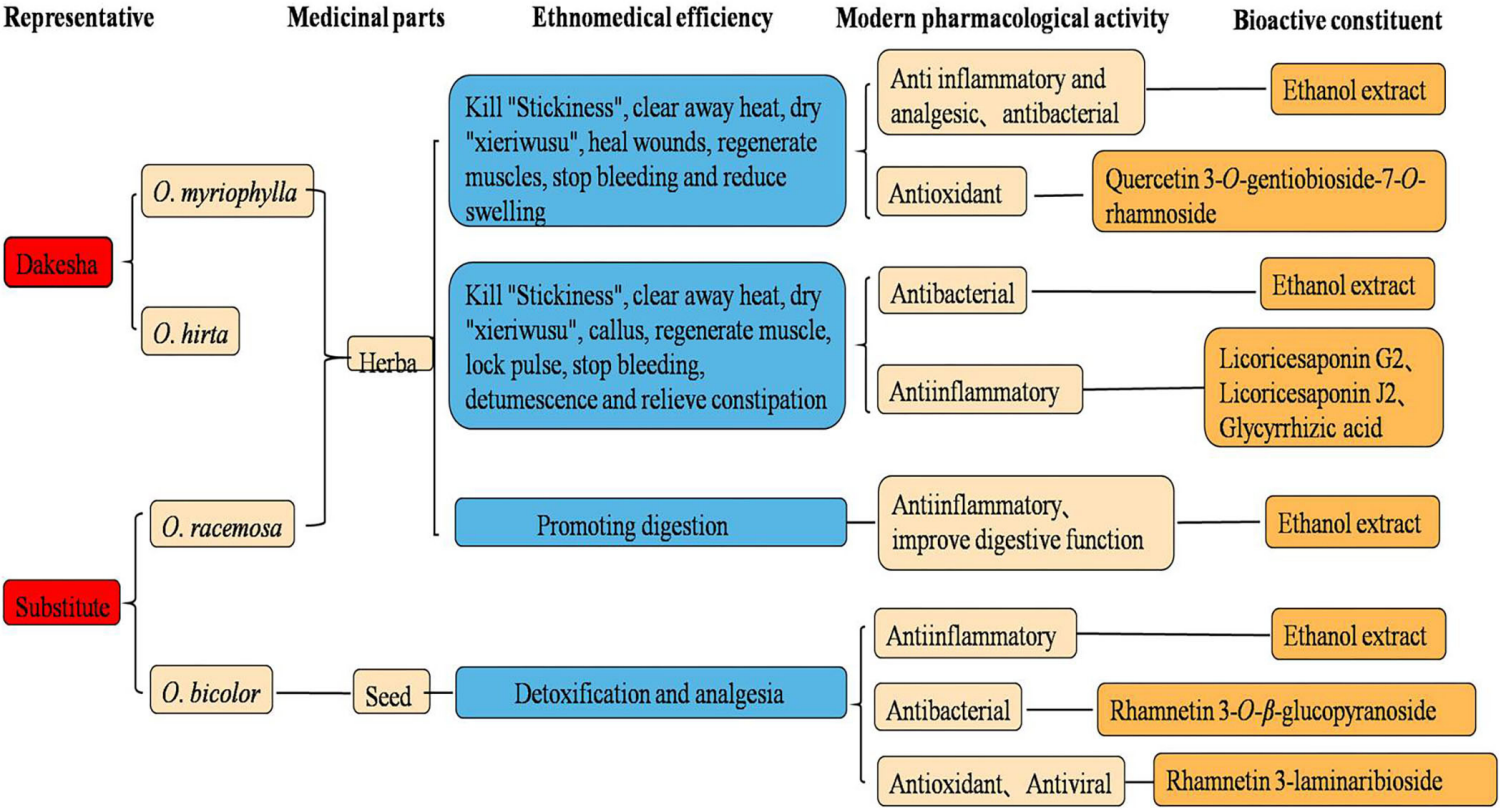
Proposed relationships between ethnopharmacology, pharmacology, and bioactive constituents from *Oxytropis*.

Investigating and summarizing the ethnomedical applications, pharmacological activities, and chemical components of the four *Oxytropis* species showed that there was a good correlation between the pharmacological activities and the ethnomedical applications of the four plant species, with many pharmacological activities having a corresponding material basis (Figure 12). At the same time, it also suggested to us that the differences in efficacies between the *Oxytropis* species may be related to their specific components. Recent research has confirmed the pharmacological efficacies of both *O. myriophylla* and *O. hirta*. For instance, *O. myriophylla* has anti-inflammatory, analgesic, antibacterial, and antioxidant effects while *O. hirta* has only antibacterial and anti-inflammatory effects. In addition, the pharmacological experiments of this study also preliminarily confirmed that the total extracts of both plants had good anti-inflammatory effects. This is closely related to the flavonoids and saponins they contain. Glycyrrhizic acid, licorice saponins J2, and licoricesaponin G2 are marker compounds specific to *O. hirta* which have been demonstrated to have anti-inflammatory effects. The effect of *O. racemosa* on digestion and strengthening the spleen has also demonstrated a relationship with pharmacological research on improving digestive function, but its material basis is not clear. In addition, the antibacterial, anti-inflammatory, antioxidant, and anti-viral activities of *O. bicolor* are mostly related to flavonoids. According to the records, *O. myriophylla* and *O. hirta* have similar traditional applications, specifically, killing “viscosity”, clearing heat, drying “xieriwusu”, healing, muscle regeneration, and hemostasis. *O. racemosa* is used for digestion and spleen strengthening, and *O. bicolor* for detoxification and analgesia. From the perspective of pharmacological activity and traditional applications, *O. myriophylla* and *O. hirta* can be used instead. To fully tap and make use of the medicinal value of this genus, further investigation on the chemical composition and activities of the components is required along with an understanding of its material basis.

## Conclusion

*Oxytropis* species are rich in resources and are widely distributed throughout the world. Although plants belonging to this genus have significant medicinal value, there has been, to date, little in-depth research on them, specifically, in the areas of classification, chemical composition, and pharmacological activities. The present study undertook to investigate its medicinal potential, summarizing the taxonomy of the genus based on relevant research and the literature. From the perspective of plant taxonomy, *O. myriophylla, O. hirta*, and *O. bicolor* are more closely related. UPLC-Q-TOF/MS combined with PCA and OPLS-DA multivariate statistical analysis was used to characterize the overall chemical compositions of four *Oxytropis* species as well as of plants from two different geographical regions. Twenty-three differential metabolites were identified, with specific metabolic markers found in the four species. An analysis of pharmacological activities showed that, compared with two other *Oxytropis* species, extracts of *O. Myriophylla* and *O. hirta* had stronger anti-inflammatory activity. The study analyzed the relationships between the specific chemical components, traditional applications, and pharmacological activities to present a preliminary study of the pharmacophylogenetics of the *Oxytropis* genus as a whole. The study showed that *O. myriophylla* and *O. hirta* are more closely related to each other than to *O. bicolor* and *O. racemose*, suggesting that it may not be effective to use the latter two species as substitutes in Mongolian medicine. This preliminary investigation of the pharmacophylogeny of the genus *Oxytropis* will contribute to the better exploitation of the medicinal potential of this genus.

## Data availability statement

The original contributions presented in the study are included in the article/Supplementary material, further inquiries can be directed to the corresponding author.

## Author contributions

XQW and XJ designed the experiment. XJ, SW, JNM, JY, XY, and YZ all performed the experiment work and prepared figures and tables. XJ and YL edited the final version of the manuscript. All the authors have studied and approved the final manuscript.

## Funding

This work was supported by grants from the National Natural Science Foundation of China (Grant Nos. 81760686), Natural Science Foundation of Inner Mongolia (Grant Nos. 2018BS08011), and Team Project Foundation of Inner Mongolia Medical University (QNLC-2020048).

## Conflict of interest

The authors declare that the research was conducted in the absence of any commercial or financial relationships that could be construed as a potential conflict of interest.

## Publisher's note

All claims expressed in this article are solely those of the authors and do not necessarily represent those of their affiliated organizations, or those of the publisher, the editors and the reviewers. Any product that may be evaluated in this article, or claim that may be made by its manufacturer, is not guaranteed or endorsed by the publisher.
