# Supplementary material for: Screening of metabolic markers present in Oxytropis by UHPLC-Q-TOF/MS and preliminary pharmacophylogenetic investigation
Source: Front Plant Sci. 2022 Oct 20;13:958460. doi: 10.3389/fpls.2022.958460 (PMC9631219; doi:10.3389/fpls.2022.958460)
Supplement: Supplementary file 1 [file Data_Sheet_1.docx]

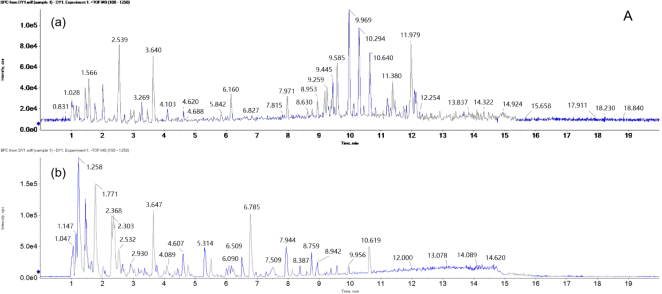


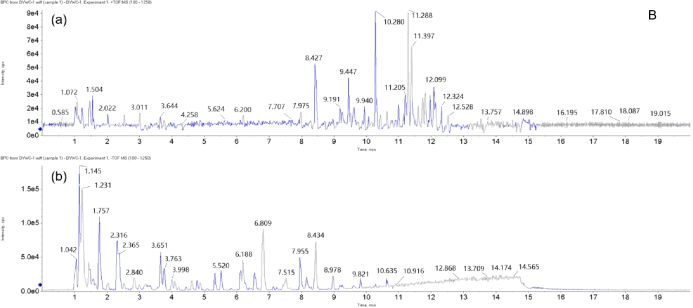


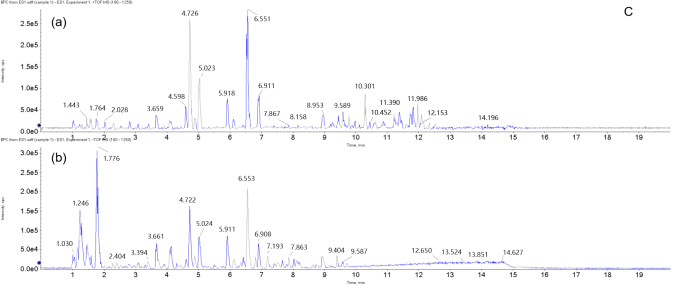


**
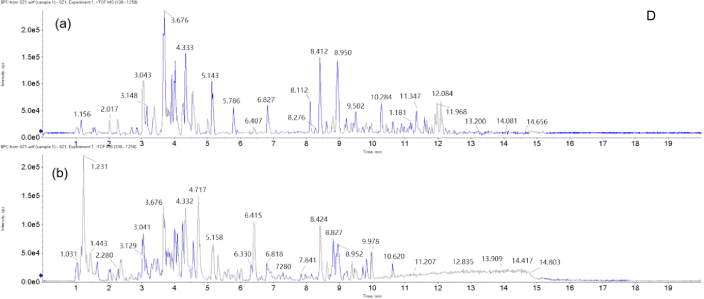
**

**
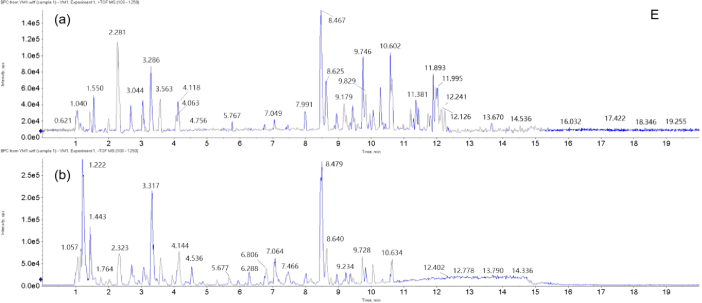
**

**FIGURE S1** UHPLC–Q-TOF–MS ion mode (a.positive mode ; b. negative mode ) in base peak ion chromatogram (BPI) diagram of ***Oxytropis*** sample (A. DW; B. DYWC; C. ES; D. SZ; E. YM)

**FIGURE S2** SVM model of *Oxytropis*

**

**FIGURE S3** SVM model of *O. myriophylla* from the two different geographical origins
