# Supplementary material for: Screening of metabolic markers present in Oxytropis by UHPLC-Q-TOF/MS and preliminary pharmacophylogenetic investigation
Source: Front Plant Sci. 2022 Oct 20;13:958460. doi: 10.3389/fpls.2022.958460 (PMC9631219; doi:10.3389/fpls.2022.958460)
Supplement: Supplementary file 2 [file Data_Sheet_2.docx]

**Table S1 Information of compounds in *Oxytropis.***

| No | Identification | tR(min) | Formula | [M-H]- | | | Fragment ions(m/z) | [M+H]+ | | | Fragment ions(m/z) | Class | Source |
| --- | --- | --- | --- | --- | --- | --- | --- | --- | --- | --- | --- | --- | --- |
|  |  |  |  | m/z | Calcm/z | Diff |  | m/z | Calcm/z | Diff |  |  |  |
|  |  |  |  |  |  | (ppm) |  |  |  | (ppm) |  |  |  |
| 1 | DL-phenylalanine | 1.562 | C_9_H_11_NO_2_ | 164.0727 | 164.0717 | 6.09 | 147,103,72 | 166.0864 | 166.0863 | 0.60 | 120, 103, 91, 77 | Amino acid | DY, ES, SZ, YM |
| 2 | proline | 1.157 | C_5_H_9_NO_2_ | - | - | - | - | 116.0706 | 116.0700 | 5.17 | 70 | Amino acid | DY |
| 3 | homocitric acid | 1.221 | C_7_H_10_O_7_ | 205.0364 | 205.0354 | 4.88 | 187, 145, 125, 101, 81, 71 | - | - | - | - | Organic acid | DY, ES, SZ, YM |
| 4 | DL-alanyl-DL-leucine | 1.709 | C_9_H_18_N_2_O_3_ | - | - | - | - | 203.1392 | 203.1390 | 0.98 | 186, 130, 84, 74 | Amino acid | DY, ES, SZ, YM |
| 5 | monoethyl citrate | 1.760 | C_8_H_12_O_7_ | 219.0517 | 219.0510 | 3.2 | 187, 159, 143, 125 | 221.0657 | 221.0656 | 0.45 | 171,157,143,125,115,97,55 | Organic acid | DY, ES, YM |
| 6 | quercetin 3-*O*-gentiobioside-7-*O*-rha | 1.781 | C_33_H_40_O_21_ | - | - | - | - | 773.2135 | 773.2135 | 0.00 | 611, 449,303 | Flavonoid glycoside | DY |
| 7 | kaempferol 3-(2gal-glucosylrobinobioside)-7-rha | 2.010 | C_39_H_50_O_24_ | 901.2626 | 901.2619 | 0.78 | 593, 284 | 903.2767 | 903.2765 | 0.22 | 757,595,433,287 | Flavonoid glycoside | YM |
| 8 | (±)-tryptophan | 2.027 | C_11_H_12_N_2_O_2_ | - | - | - | - | 205.0976 | 205.0972 | 1.95 | 188, 170, 146, 115, 91, 77 | Amino acid | DY, ES, SZ, YM |
| 9 | kaempferol 3-gentiobioside-7-rha | 2.036 | C_31_H_60_O_33_ | 755.2032 | 755.2040 | -1.06 | 609284 | 757.2179 | 757.2186 | -0.92 | 595, 433, 287 | Flavonoid glycoside | DY |
| 10 | salicylic acid glucoside | 2.168 | C_13_H_16_O_8_ | 299.0786 | 299.0772 | 4.68 | 137,93 | - | - | - | - | Glucoside | DY, ES, SZ, YM |
| 11 | robinin | 2.322 | C_33_H_40_O_19_ | 739.209 | 739.2091 | -0.14 | 593,430,284 | 741.2236 | 741.2237 | -0.13 | 595, 433, 287 | Flavonoid glycoside | YM |
| 12 | quercetin 3-(6''-caffeoylsophoroside)-7-rha | 2.495 | C_42_H_46_O_24_ | 933.2313 | 933.2306 | 0.75 | 787, 771, 625, 446, 300 | 935.2456 | 935.2452 | 0.43 | 449,303,163 | Flavonoid glycoside | DY |
| 13 | unknown | 2.510 | C_35_H_42_O_22_ | - | - | - | - | 815.2240 | 815.2239 | -0.12 | 815,653,507,449,303 | Flavonoid glycoside | DY |
| 14 | Thr-leu | 2.519 | C_10_H_20_N_2_O_4_ | - | - | - | - | 233.1496 | 233.1499 | -1.29 | 216,198,169,146,88,82,71 | Amino acid | DY |
| 15 | quercetin-3rha-gdl | 2.720 | C_45_H_48_O_27_ | - | - | - | - | 1021.245 | 1021.246 | 0.98 | 875, 697, 551, 449, 411, 325, 307, 303 | Flavonoid glycoside | DY |
| 16 | kaempferol 3-(2''-caffeyllaminaribioside)-7-rhamnoside/kaempferol 3-(4''-caffeyllaminaribioside)-7-rha | 2.780 | C_42_H_46_O_23_ | 917.2367 | 917.2357 | 1.09 | 917, 771, 609,285, 161 | 919.2507 | 919.2499 | 0.87 | 595,433,287,163 | Flavonoid glycoside | DY |
| 17 | quercetin-2glc-2rha | 2.968 | C_44_H_48_O_25_ | 975.2418 | 975.2412 | 0.62 | 829,813,667,446 | 977.2557 | 977.2596 | -3.99 | 653,449,325,303,163 | Flavonoid glycoside | DY |
| 18 | sarmenoside III | 3.015 | C_42_H_46_O_23_ | 917.2363 | 917.2357 | 0.65 | 917, 771, 625, 446, 300 | 919.2507 | 919.2503 | 0.44 | 449,303,147 | Flavonoid glycoside | DY |
| 19 | kaempferol 3-(2''-rhamnosyl-6''-acetylgalactoside) 7-rha | 3.039 | C_35_H_42_O_20_ | 781.2198 | 781.2197 | 0.13 | 635430284 | 783.2338 | 783.2342 | -0.51 | 637, 433, 287, 187 | Flavonoid glycoside | YM |
| 20 | isorhamnetin 3-(2G-apiosylrutinoside)/isorhamnetin 3-*O*-[*β*-D-xyl-(1→2)-[*α*-L-rha-(1→6)]-*β*-D-glucopyranoside] | 3.062 | C_33_H_40_O_20_ | 755.2062 | 755.2040 | 2.91 | 315,314,299,271 | 757.2186 | 757.2196 | -1.32 | 479,317 | Flavonoid glycoside | SZ |
| 21 | quercetin-rha-glc-glc(propanoic acid)-rha | 3.190 | C_45_H_48_O_26_ | - | - | - | - | 1005.251 | 1005.252 | 0.99 | 1005,859, 697,557,449,303,147 | Flavonoid glycoside | DY |
| 22 | 3-(*β*-D-glucopyranosyloxy)-4',5-dihydroxy-7-[2-*O*-[6-*O*-(3-methoxy-4-hydroxy-trans-cinnamoyl)-*β*-D-glu]-*α*-L-rhamnopyranosyloxy]flavone | 3.256 | C_43_H_48_O_23_ | 931.2498 | 931.2514 | -1.72 | 785,609,284 | 933.2666 | 933.2659 | 0.75 | 433, 287, 177 | Flavonoid glycoside | DY |
| 23 | kaempferol 3-caffeylrobinobioside-7-rha | 3.258 | C_42_H_46_O_22_ | 901.2423 | 901.2408 | 1.66 | 901, 755, 609, 284 | 903.2559 | 903.2554 | 0.55 | 433,287,147 | Flavonoid glycoside | DY |
| 24 | unknown | 3.323 | C_15_H_19_NO_8_ | 340.1045 | 340.1038 | 2.06 | 161, 101 | - | - | - | - | Glucoside | YM |
| 25 | quercetin-3rha-glu | 3.446 | C_44_H_48_O_24_ | 959.2454 | 959.2463 | -0.94 | 300 | 961.2608 | 961.2614 | -0.62 | 961,653,449,303,149 | Flavonoid glycoside | DY |
| 26 | kaempferol-rha-glu-glu(propanoic acid)-rha | 3.449 | C_45_H_48_O_25_ | 987.2414 | 987.2412 | 0.2 | 987, 943, 797, 651, 284 | 989.2563 | 989.2557 | 0.61 | 681,433,287,147 | Flavonoid glycoside | DY |
| 27 | quercetin-3rha-gluA | 3.450 | C_45_H_48_O_26_ | 1003.236 | 1003.2360 | 0 | 959,813,667,446,300 | - | - | - | - | Flavonoid glycoside | DY |
| 28 | unknown | 3.470 | C_47_H_50_O_28_ | - | - | - | - | 1063.256 | 1063.257 | 0.94 | 913,653,449,303,163 | Flavonoid glycoside | DY |
| 29 | unknown | 3.480 | C_46_H_50_O_26_ | - | - | - | - | 1019.266 | 1019.268 | 1.96 | 873,681,587, 433,339,287,177,145 | Flavonoid glycoside | DY |
| 30 | kaempferol-rha-gluA-2glu | 3.490 | C_44_H_46_O_24_ | 1017.252 | 1017.2500 | -1.97 | 973,827,651,284 | - | - | - | - | Flavonoid glycoside | DY |
| 31 | arbortristoside E | 3.658 | C_27_H_34_O_13_ | 565.1925 | 565.1927 | -0.35 | 339, 327, 324 | - | - | - | - | Iridoid glycoside | DY, ES, SZ, YM |
| 32 | isorhamnetin 3-*O*-[*β*-D-xyl-(1→6)-*β*-D-glu] | 3.680 | C_27_H_30_O_16_ | 609.1467 | 609.1461 | 0.98 | 315 | 611.1607 | 611.1607 | 0.00 | 317 | Flavonoid glycoside | SZ |
| 33 | quercetin 3-*O-β*-glu | 3.685 | C_21_H_20_O_12_ | 463.087 | 463.0858 | 2.59 | 301,300 | 465.1028 | 465.1028 | 0.00 | 303 | Flavonoid glycoside | ES, DY |
| 34 | kaempferol 3-glucuronide | 3.748 | C_21_H_18_O_12_ | 461.072 | 461.0725 | -1.08 | 461,285 | - | - | - | - | Flavonoid glycoside | DY,YM |
| 35 | kaempferol 3-*O*-(2''-*O-α*-rha-6''-*O*-malonyl)-*β*-D-glu | 3.947 | C_30_H_32_O_18_ | 679.1512 | 679.1492 | 2.94 | 635,430,285 | 681.1666 | 681.1661 | 0.73 | 535, 287 | Flavonoid glycoside | YM |
| 36 | indoleacetylaspartate | 4.071 | C_14_H_14_N_2_O_5_ | - | - | - | - | 291.0979 | 291.0975 | 1.37 | 245, 185, 132 | Alkaloid | DY, ES, SZ, YM |
| 37 | cynaroside | 4.138 | C_28_H_16_O_6_ | - | - | - | - | 449.1078 | 449.1078 | 0.00 | 287 | Flavonoid glycoside | ES, YM |
| 38 | isorhamnetin3-*O-β*-glu | 4.275 | C_22_H_22_O_12_ | 477.104 | 477.1039 | 0.21 | 315,285,271,243,151 | 479.1186 | 479.1184 | 0.42 | 317 | Flavonoid glycoside | SZ |
| 39 | unknown | 4.380 | C_17_H_20_N_2_O_6_ | - | - | - | - | 349.1394 | 349.1397 | 0.86 | 267, 188, 146, 118 | Alkaloid | DY |
| 40 | unknown | 4.450 | C_34_H_44_O_15_ | 691.2607 | 691.2616 | 1.3019673 | 335,317 | - | - | - | - | Flavonoid glycoside | DY |
| 41 | rhamnetin 3-sophoroside/isorhamnetin 3-laminaribioside/rhamnetin 3-laminaribioside | 4.595 | C_28_H_32_O_17_ | 639.1565 | 639.1567 | -0.31 | 477,315,165 | 641.1717 | 641.1712 | 0.78 | 479, 317 | Flavonoid glycoside | ES |
| 42 | rhamnetin or isorhamnetin 3-glu-malonyl | 4.662 | C_25_H_24_O_15_ | 563.1057 | 563.1042 | 2.66 | 519,314,315 | 565.1192 | 565.1188 | 0.71 | 317 | Flavonoid glycoside | SZ |
| 43 | chrysoeriol-7-*O*-(2''-*O*-mannopyranosyl)allopyranoside/complanatuside | 4.697 | C_28_H_32_O_16_ | - | - | - | - | 625.1768 | 625.1763 | 0.80 | 463, 301 | Flavonoid glycoside | ES |
| 44 | [(2R,3R)-2-(3,4-dihydroxyphenyl)-5,7-dihydroxy-3,4-dihydro-2H-chromen-3-yl]oxymethyl hydrogen carbonate | 4.698 | C_17_H_16_O_9_ | 363.0726 | 363.0722 | 1.1 | 345, 299, 285, 271, 151 | - | - | - | - | Flavonoid glycoside | SZ |
| 45 | 3-[6-*O*-[4-*O*-(4-Oxo-4-hydroxybutyryl)-6-deoxy-*α*-L-mannopyranosyl]-*β*-D-glu]-3',4',5,7-tetrahydroxyflavone | 5.007 | C_31_H_34_O_19_ | 709.1609 | 709.1622 | -1.83 | 503,461,299 | 711.1767 | 711.1767 | 0.00 | 463, 301 | Flavonoid glycoside | ES |
| 46 | unknown | 5.784 | C_22_H_23_NO_9_ | 444.1299 | 444.1300 | -0.23 | 282,131 | 446.1445 | 446.1446 | -0.22 | 446, 284, 269, 134 | Alkaloid | SZ |
| 47 | rhamnetin3-*O-β*-glu | 5.927 | C_22_H_22_O_12_ | 477.1031 | 477.1039 | -1.68 | 315,299,271,165 | 479.1183 | 479.1184 | -0.21 | 317 | Flavonoid glycoside | ES |
| 48 | irisxanthone | 6.007 | C_20_H_20_O_11_ | 435.0934 | 435.0933 | 0.23 | 315, 297, 137 | - | - | - | - | Flavonoid glycoside | DY, ES, SZ, |
| 49 | soyasaponin A3 | 6.414 | C_48_H_78_O_19_ | 957.5069 | 957.5065 | 0.42 | - | 959.5217 | 959.5210 | 0.73 | 813, 651, 633, 615, 457 | Saponin | SZ, ES |
| 50 | thermopsoside/kaempferide 7-glucoside | 6.550 | C_22_H_22_O_11_ | 461.1083 | 461.1089 | -1.3 | 299,284,255,227 | 463.1236 | 463.1235 | 0.22 | 301 | Flavonoid glycoside | ES |
| 51 | triterpenoid-xyl-rha-gluA | 6.831 | C_47_H_74_O_19_ | 941.4765 | 941.4752 | 1.38 | - | 943.4891 | 943.4897 | -0.64 | 943, 811, 635, 489, 471, | Saponin | SZ |
| 52 | luteolin 3'-methyl ether 7-malonylglucoside/quercetin 3-(3'',6''-diacetylgalactoside) | 6.928 | C_25_H_24_O_14_ | 547.1083 | 547.1093 | -1.83 | 503,299,284 | 549.1243 | 549.1239 | 0.73 | 301 | Flavonoid glycoside | ES |
| 53 | saponin 2 | 6.935 | C_48_H_76_O_20_ | - | - | - | - | 973.5013 | 973.5003 | 1.06 | 827, 665, 471 | Triterpene glycoside | DY, ES, SZ, YM |
| 54 | licoricesaponin G2 | 8.009 | C_42_H_62_O_17_ | 837.3906 | 837.3914 | -0.96 | 351 | 839.4068 | 839.4060 | 0.95 | 663, 645, 627, 469, 451, 439 | Saponin | YM |
| 55 | glycyrrhizic acid | 8.427 | C_42_H_62_O_16_ | 821.3944 | 821.3965 | -2.56 | 759, 645, 351, 289, 193, 113 | 840.4375  [M+NH_4_]^+^ | 840.4376 | -0.12 | 451 | Saponin | YM |
| 56 | chiisanoside | 8.802 | C_48_H_74_O_19_ | 953.4746 | 953.4752 | -0.63 | - | 955.4909 | 955.4897 | 1.26 | 809, 647,611, 471, 453 | Saponin | SZ, ES |
| 57 | soyasaponin I | 8.957 | C_48_H_78_O_18_ | 941.5083 | 941.5115 | -3.4 | - | 943.5266 | 943.5261 | 0.53 | 797, 781, 635, 617, 599, 459, 441, 423 | Saponin | DY, ES, SZ, YM |
| 58 | soyasaponin III | 9.207 | C_42_H_68_O_14_ | - | - | - | - | 797.4687 | 797.4682 | 0.63 | 635, 599, 581, 441, 423, 405 | Saponin | DY, ES, SZ, YM |
| 59 | unknown | 9.430 | C_40_H_64_O_10_ | - | - | - | - | 705.4572 | 705.4572 | 0.00 | 705,687,669,439, 421,231,171 | Terpene | DY |
| 60 | dehydrosoyasaponin I | 9.529 | C_48_H_76_O_18_ | 939.4945 | 939.4959 | -1.49 | - | 941.5061 | 941.5104 | -4.57 | 941, 795, 633, 597, 439, 421 | Saponin | DY, ES, SZ, YM |
| 61 | 3-phenyl-N-(2-phenylethyl)-2-propenamide | 9.620 | C_17_H_17_NO | - | - | - | - | 252.1385 | 252.1383 | 0.79 | 131, 105 | Alkaloid | ES, YM |
| 62 | licoricesaponin J2 | 9.747 | C_42_H_64_O_16_ | 823.41 | 823.4122 | -2.67 | 351 | 825.4262 | 825.4267 | -0.61 | 825, 649, 613, 455, 437, 141 | Saponin | YM |
| 63 | pedunsaponin A/azukisaponin III | 9.849 | C_42_H_66_O_15_ | - | - | - | - | 811.4471 | 811.4474 | -0.37 | 613, 455, 437,397 | Saponin | DY, ES, SZ, YM |
| 64 | 2-(2-amino-3-methoxyphenyl)-4H-1-benzopyran-4-one | 9.969 | C_16_H_13_NO_3_ | - | - | - | - | 268.0974 | 268.0968 | 2.24 | 253, 150, 105, 77 | Alkaloid | DY, ES, SZ, YM |
| 65 | unknown | 10.283 | C_24_H_30_O_6_ | - | - | - | - | 415.2116 | 415.2115 | 0.24 | 119 | Terpene | DY, ES, SZ, YM |
| 66 | 3*β*-(*β*-D-glu)oleana-12-ene-28,30-dioic acid 30-methyl ester | 10.482 | C_37_H_56_O_11_ | 675.3753 | 675.3750 | 0.44 | 499 | 677.3892 | 677.3895 | -0.44 | 659, 501, 486, 465, 249 | Saponin | SZ |
| 67 | tragopogonsaponin A | 10.666 | C_36_H_56_O_10_ | 647.389 | 647.3801 | 13.75 | 471,423 | 666.4214[M+NH4]+ | 666.4212 | 0.30 | 455, 437 | Saponin | DY, ES, SZ, YM |
| 68 | triterpenoid-glu-gluA | 11.362 | C_43_H_68_O_15_ | 823.4497 | 823.4485 | 1.46 | - | 825.4634 | 825.4631 | 0.36 | 825, 663, 645, 627, 469, 451 | Saponin | DY, SZ |
| 69 | citric acid | 1.190/1.442 | C_6_H_8_O_7_ | 191.0209 | 191.0197 | 6.28 | 129,111,87,85,67 | - | - | - | - | Organic acid | DY, ES, SZ, YM |
| 70 | luteolin-7-*O*-rha/ kaempferol-3-*O*-rutinoside/kampferol 3-glu7-rha | 2.301/3.054/3.579 | C_27_H_30_O_15_ | 593.151 | 593.1512 | -0.34 | 284 | 595.1652 | 595.1658 | -1.01 | 433, 287 | Flavonoid glycoside | YM |
| 71 | herbacetin 7-(6''-quinoylglucoside) | 3.312/3.738 | C_28_H_30_O_17_ | 637.1402 | 637.1410 | -1.26 | 637, 595, 301, 300 | - | - | - | - | Flavonoid glycoside | SZ |
| 72 | 3-[4-*O*-(6-*O*-Acetyl-*β*-D-glu)-*α-*L-rha]-4',5,7-trihydroxyflavone/kaempferol 3-*O*-(6''-*O*-acetyl)glucoside-7-*O*-rha/multiflorin A | 3.369/3.682/3.958/4.514 | C_29_H_32_O_16_ | 635.1625 | 635.1618 | 1.1 | 284, 255 | 637.1759 | 637.1763 | -0.63 | 433,287 | Flavonoid glycoside | YM |
| 73 | isorhamnetin 3-(2'''-acetyl-*α*-arabinopyranosyl)-(1→6)-galactoside/tricetin 4'-methyl ether 7-apiosyl-(1→2)-(6''-acetylglucoside) | 3.901/4.032/4.336 | C_29_H_32_O_17_ | 651.1578 | 651.1567 | 1.69 | 315,300 | 653.1716 | 653.1712 | 0.61 | 315, 175, 157 | Flavonoid glycoside | SZ |
| 74 | unknown | 4.555/5.147 | C_31_H_34_O_18_ | 693.1685 | 693.1672 | 1.88 | 315 | 695.1829 | 695.1818 | 1.58 | 317, 217, 97 | Flavonoid glycoside | SZ |
| 75 | amaranthussaponin I | 7.288/7.494 | C_48_H_76_O_19_ | 955.4896 | 955.4908 | -1.26 | - | 957.5081 | 957.5054 | 2.82 | 811, 649, 613, 473, 455 | Saponin | ES, SZ |

**Table S2 Discrepant components of *O. myriophylla* of two different geographical origins**

| NO. | Rt(min) | Compound | Formula | VIP | pcorr | P | Fold |
| --- | --- | --- | --- | --- | --- | --- | --- |
| 1 | 1.48 | homocitric acid | C_7_H_10_O_7_ | 8.23 | -0.68 | 0.02 | 0.53 |
| 2 | 4.65 | byzantionoside B | C_19_H_32_O_7_ | 5.94 | -0.91 | 0.00 | 0.27 |
| 3 | 3.48 | kaempferol-rha-glu-glu(propanoic acid)-rha | C_45_H_48_O_25_ | 5.62 | -0.93 | 0.00 | 0.11 |
| 4 | 4.45 | unknown | C_34_H_44_O_15_ | 5.38 | -0.84 | 0.00 | 0.45 |
| 5 | 3.45 | quercetin-3rha-gluA | C_45_H_48_O_26_ | 4.40 | -0.85 | 0.00 | 0.16 |
| 6 | 3.49 | kaempferol-rha-gluA-2glu | C_44_H_46_O_24_ | 4.18 | -0.93 | 0.00 | 0.17 |
| 7 | 2.54 | Thr-leu | C_10_H_20_N_2_O_4_ | 10.83 | -0.83 | 0.00 | 0.27 |
| 8 | 2.98 | quercetin-2glu-2rha | C_44_H_48_O_25_ | 10.51 | -0.77 | 0.00 | 0.01 |
| 9 | 9.97 | 2-(2-amino-3-methoxyphenyl)-4H-1-benzopyran-4-one | C_16_H_13_NO_3_ | 10.38 | -0.61 | 0.04 | 0.20 |
| 10 | 2.72 | quercetin-3rha-gdl | C_45_H_48_O_27_ | 8.77 | -0.79 | 0.00 | 0.06 |
| 11 | 10.64 | tragopogonsaponin A | C_36_H_56_O_10_ | 8.14 | -0.74 | 0.01 | 0.40 |
| 12 | 2.51 | quercetin 3-(6''-caffeoylsophoroside)-7-rha | C_42_H_46_O_24_ | 7.90 | -0.80 | 0.00 | 0.26 |
| 13 | 3.19 | quercetin-rha-glu-glu(propanoic acid)-rha | C_45_H_48_O_26_ | 7.77 | -0.82 | 0.00 | 0.18 |
| 14 | 4.11 | indoleacetylaspartate | C_14_H_14_N_2_O_5_ | 5.10 | -0.85 | 0.00 | 0.57 |
| 15 | 2.51 | unknown | C_35_H_42_O_22_ | 4.86 | -0.92 | 0.00 | 0.04 |
| 16 | 9.87 | pedunsaponin A/azukisaponin III | C_42_H_66_O_15_ | 4.68 | -0.75 | 0.00 | 0.34 |
| 17 | 3.47 | unknown | C_47_H_50_O_28_ | 4.66 | -0.82 | 0.00 | 0.03 |
| 18 | 4.37 | phellodensin F | C_26_H_30_O_10_ | 4.14 | -0.80 | 0.00 | 0.04 |
| 19 | 1.18 | proline | C_5_H_9_NO_2_ | 3.87 | -0.90 | 0.00 | 0.70 |
| 20 | 3.48 | unknown | C_46_H_50_O_26_ | 3.87 | -0.82 | 0.00 | 0.17 |
| 21 | 9.43 | unknown | C_40_H_64_O_10_ | 3.60 | -0.80 | 0.00 | 0.10 |
| 22 | 4.38 | unknown | C_17_H_20_N_2_O_6_ | 3.26 | -0.80 | 0.00 | 0.02 |
| 23 | 3.44 | 6''-acetylhyperin 7-rha | C_29_H_32_O_17_ | 2.79 | -0.83 | 0.00 | 0.04 |
| 24 | 1.57 | DL-phenylalanine | C_9_H_11_NO_2_ | 5.78 | -0.86 | 0.00 | 0.71 |
| 25 | 2.77 | kaempferol 3-caffeylrobinobioside-7-rha | C_42_H_46_O_22_ | 1.12 | -0.72 | 0.01 | 0.52 |
| 26 | 4.21 | arbortristoside E | C_27_H_34_O_13_ | 4.76 | -0.68 | 0.02 | 0.37 |
